# GSAP regulates lipid homeostasis and mitochondrial function associated with Alzheimer’s disease

**DOI:** 10.1084/jem.20202446

**Published:** 2021-06-22

**Authors:** Peng Xu, Jerry C. Chang, Xiaopu Zhou, Wei Wang, Michael Bamkole, Eitan Wong, Karima Bettayeb, Lu-Lin Jiang, Timothy Huang, Wenjie Luo, Huaxi Xu, Angus C. Nairn, Marc Flajolet, Nancy Y. Ip, Yue-Ming Li, Paul Greengard

**Affiliations:** 1 Laboratory of Molecular and Cellular Neuroscience, The Rockefeller University, New York, NY; 2 Chemical Biology Program, Memorial Sloan Kettering Cancer Center, New York, NY; 3 Division of Life Science, State Key Laboratory of Molecular Neuroscience and Molecular Neuroscience Center, The Hong Kong University of Science and Technology, Hong Kong, China; 4 Hong Kong Center for Neurodegenerative Diseases, Hong Kong Science and Technology Parks, Hong Kong, China; 5 Guangdong Provincial Key Laboratory of Brain Science, Disease, and Drug Development, Shenzhen–Hong Kong Institute of Brain Science, HKUST Shenzhen Research Institute, Shenzhen, Guangdong, China; 6 Neuroscience Initiative, Sanford Burnham Prebys Medical Discovery Institute, La Jolla, CA; 7 Brain and Mind Research Institute, Weill Cornell Medical College, New York, NY; 8 Department of Psychiatry, Yale School of Medicine, Connecticut Mental Health Center, New Haven, CT; 9 Program of Pharmacology and Neurosciences, Weill Graduate School of Medical Sciences of Cornell University, New York, NY

## Abstract

Biochemical, pathogenic, and human genetic data confirm that GSAP (γ-secretase activating protein), a selective γ-secretase modulatory protein, plays important roles in Alzheimer’s disease (AD) and Down’s syndrome. However, the molecular mechanism(s) underlying GSAP-dependent pathogenesis remains largely elusive. Here, through unbiased proteomics and single-nuclei RNAseq, we identified that GSAP regulates multiple biological pathways, including protein phosphorylation, trafficking, lipid metabolism, and mitochondrial function. We demonstrated that GSAP physically interacts with the Fe65–APP complex to regulate APP trafficking/partitioning. GSAP is enriched in the mitochondria-associated membrane (MAM) and regulates lipid homeostasis through the amyloidogenic processing of APP. GSAP deletion generates a lipid environment unfavorable for AD pathogenesis, leading to improved mitochondrial function and the rescue of cognitive deficits in an AD mouse model. Finally, we identified a novel GSAP single-nucleotide polymorphism that regulates its brain transcript level and is associated with an increased AD risk. Together, our findings indicate that GSAP impairs mitochondrial function through its MAM localization and that lowering GSAP expression reduces pathological effects associated with AD.

## Teaser

GSAP plays an important role in Alzheimer's disease, but the underlying cellular and molecular pathways remain unclear. Using unbiased multi-omics approaches, this study demonstrates that GSAP regulates lipid homeostasis in mitochondria-associated membranes (MAM), which affects mitochondrial function associated with the disease.

## Introduction

γ-Secretase activating protein (GSAP) plays an important role in regulating γ-secretase activity and specificity. GSAP selectively modulates γ-secretase activity toward amyloid precursor protein (APP) cleavage, but not Notch (He et al., 2010; Wong et al., 2019). Depletion of GSAP consistently decreases amyloid-β (Aβ) generation in cells (He et al., 2010; Hussain et al., 2013; Wong et al., 2019). Furthermore, genetic knockdown or pharmacological inhibition of GSAP lowers amyloid plaque deposition and tau phosphorylation in AD mouse models (Chu et al., 2014; Chu et al., 2015; He et al., 2010). Recently, it has been reported that GSAP physically interacts with APP to regulate Aβ generation (Angira et al., 2019). In addition to increased GSAP levels observed in AD mouse models (Chu et al., 2015), GSAP up-regulation has also been reported in neurodegenerative contexts such as Down’s syndrome (Chu et al., 2016), which is obligately associated with Aβ plaque pathology due to triplication of human chromosome 21 harboring APP (Wiseman et al., 2015). Importantly, several studies have also independently demonstrated that GSAP levels are significantly increased in postmortem brains of severe AD patients (Chu et al., 2015; Perez et al., 2017; Satoh et al., 2012). Single-nucleotide polymorphisms (SNPs) at the GSAP locus have also been identified and have been shown to correlate with AD diagnosis (Floudas et al., 2014; Zhu et al., 2014). One SNP located in the GSAP promoter region comprises an allele associated with high GSAP expression, which correlates with increased AD risk (Zhu et al., 2014). Together, these studies implicate a pathogenic role for GSAP in AD. Aside from its role in activating γ-secretase activity and APP trafficking/partitioning, little is known about other biological pathways involved in GSAP-dependent AD pathogenesis.

In this study, we identified novel GSAP-binding proteins by proteomic analysis and demonstrate that GSAP regulates APP phosphorylation and trafficking/partitioning through physical interactions with the APP-binding protein Fe65. We also compared transcriptomic profiles in WT and GSAP KO (GKO) mouse hippocampus by single-nuclei RNA sequencing (sn-RNAseq). Pathway enrichment analysis of proteomic and sn-RNAseq datasets concordantly identified overlapping biological pathways associated with GSAP, including protein phosphorylation, trafficking, lipid metabolism, and mitochondrial function. We further demonstrated that GSAP is enriched in the mitochondria-associated membrane (MAM) and promotes APP C-terminal fragment (CTF) partitioning into lipid rafts in favor of Aβ production. We demonstrated that GSAP deletion changed the cellular lipid profile and restored impaired memory behavior by novel object recognition tests in the J20 AD mouse model. Finally, a novel SNP was identified and shown to specifically regulate GSAP mRNA expression in human brain; the allele associated with high GSAP expression was found to correlate with AD risk. Taken together, our findings uncover new pathogenic pathways mediated by GSAP and provide evidence that reducing GSAP levels can attenuate pathogenic events associated with AD.

## Results

### The GSAP complex regulates protein phosphorylation, trafficking, lipid metabolism, and mitochondrial function

**Figure 1.**
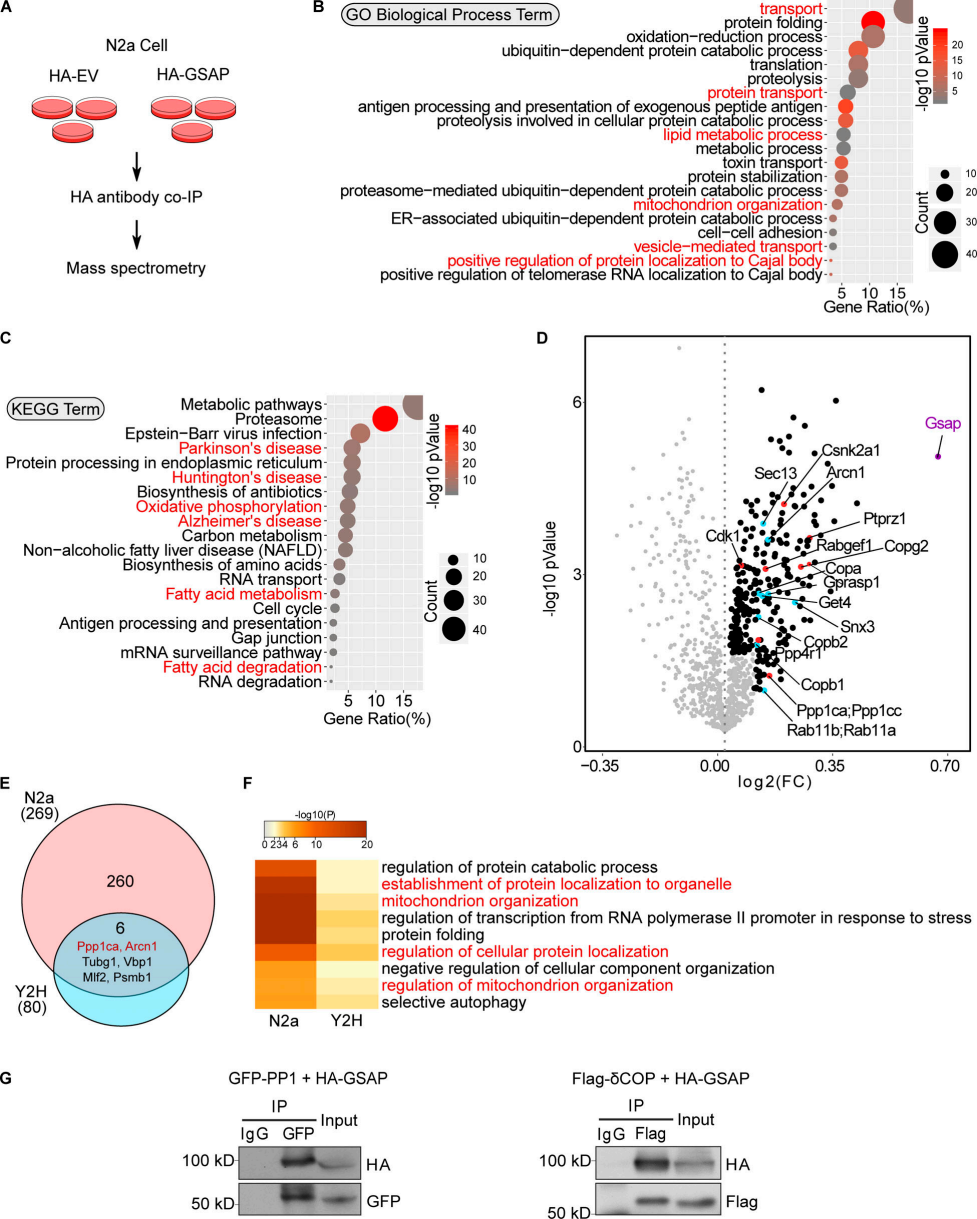
**GSAP and its binding proteins are involved in novel biological pathways. (A)** Schematic of the experimental design to characterize the GSAP interactome. HA-EV was used as a negative control. **(B)** GO pathway enrichment analysis for GSAP-binding proteins. Top 20 significantly enriched pathways (P < 0.05) are shown based on P value (dot color) and gene count (dot size). **(C)** KEGG biological process enrichment analysis for GSAP-binding proteins. Top 20 significantly enriched pathways (P < 0.05) are shown based on P value (dot color) and gene count (dot size). **(D)** Volcano plot showing differentially enriched proteins (detailed in the methods) in HA-GSAP versus HA-EV co-IP MS experiments in N2a cells. GSAP itself (purple), proteins involved in trafficking (blue), and phosphorylation (red) are highlighted. FC, fold change. **(E)** Venn diagram showing overlapped protein between different lists. The circle area is not proportional to the sample size. **(F)** Meta-enrichment analysis of common GO biological pathways shared by two GSAP-binding protein lists. **(G)** Co-IP validation of GSAP interaction with PP1 and δ-COP (Arcn1) in HEK293T or N2a cells, respectively, via transient transfection. Representative data of three experiments.

**Figure S1.**
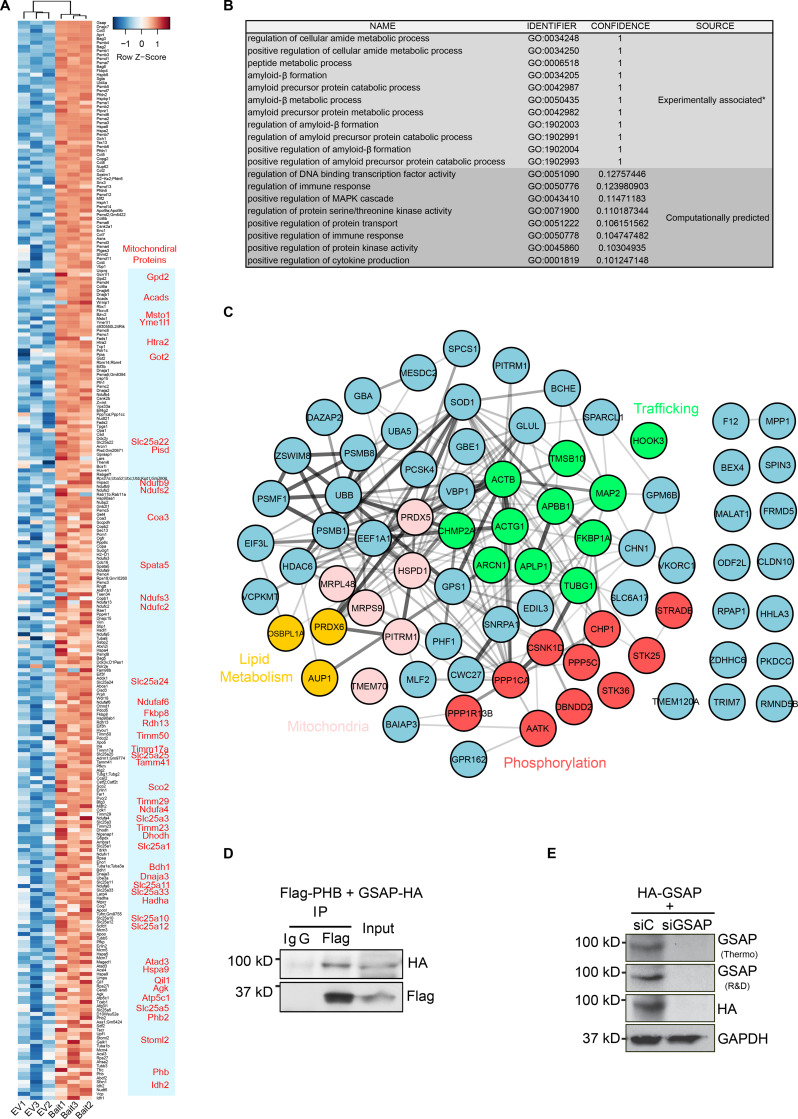
**GSAP-binding protein and antibody validation. (A)** Heatmap showing GSAP and binding protein levels in bait-expressing (HA-GSAP) versus EV (empty vector expression) samples in N2a co-IP and MS analyses. Proteins enriched in HA-GSAP samples are shown; mitochondrial proteins are highlighted in red. **(B)** GO biological process association for GSAP from experimental data and computational prediction (humanbase database; http://hb.flatironinstitute.org/gene/54103). *, based on previous experimental data. **(C)** GSAP-binding proteins identified through Y2H were visualized by the STRING App in Cytoscape. **(D)** Co-IP analysis of GSAP (HA-tagged) interaction with PHB (Flag-tagged) using Flag antibody. Representative data of two experiments. **(E)** HA-tagged human GSAP plasmid was transfected into HEK293T cells together with control (C) or GSAP siRNA. 48 h after transfection, cell lysates were collected and subjected to SDS-PAGE and immunoblot analysis. GSAP antibody from Thermo Fisher Scientific (Thermo) or R&D Systems (R&D) was used to detect GSAP. Representative data of two experiments.

To investigate new players in GSAP function, we identified GSAP-binding proteins by two approaches. We first performed coimmunoprecipitation (co-IP) from mouse neuroblastoma N2a cells followed by mass spectrometry (MS) analysis. Using N2a cells to transiently express either hemagglutinin (HA)-tagged empty vector (HA-EV) or HA-GSAP plasmids (Fig. 1 A), proteins were immunoprecipitated using the HA antibody, and proteins specifically enriched in HA-GSAP transfected samples were subjected to Kyoto Encyclopedia of Genes and Genomes (KEGG) and Gene Ontology (GO) pathway analyses (Fig. 1, B and C; and Fig. S1 A). GO pathway analysis suggested that GSAP and its binding protein complex regulate transport, lipid metabolism, and mitochondrial function, which are essential pathways altered in AD (Fig. 1 B). Interestingly, KEGG pathway analysis demonstrated that GSAP and its binding proteins may be involved in multiple neuronal disorders, including AD (Fig. 1 C). Within GSAP-binding partners, we identified multiple kinases and phosphatases (Fig. 1 D, highlighted in red), in addition to proteins directly involved in trafficking (Fig. 1 D, highlighted in blue). A significant number of mitochondrial proteins were also observed in the GSAP interactome (Fig. S1 A, highlighted in red). We also assessed the biological function of GSAP using Humanbase, a machine learning–based framework (http://hb.flatironinstitute.org/gene/54103/Biologicalprocess). In good agreement with our results here, GSAP was predicted to play essential roles in protein transport and phosphorylation regulation (Fig. S1 B). Next, we performed yeast two-hybrid (Y2H) screening of a human brain cDNA library using the 16-kD C-terminal domain of human GSAP (GSAP-16K) as bait. GSAP-16K is the functional domain responsible for γ-secretase activity regulation (He et al., 2010). We identified 80 proteins that can directly bind GSAP through the 16K domain that also may regulate phosphorylation, trafficking, lipid metabolism, and mitochondrial function (Fig. S1 C).

Direct comparison of GSAP-binding proteins identified by these two approaches uncovered six common proteins (Fig. 1 E). Meta-enrichment analysis of shared biological pathways demonstrated that protein trafficking– and mitochondria-related biological pathways were the top GO pathways shared by these two lists (Fig. 1 F). We then validated some of the GSAP-binding proteins from the lists, including PP1 (phosphorylation), prohibitin (PHB; mitochondrial function), and δCOP (encoded by the Arcn1 gene; trafficking). We confirmed that GSAP interacts with PP1 (PP1γ encoded by the Ppp1cc gene), PHB, and δCOP by co-IP analysis (Figs. 1 G and S1 D). Notably, we recently showed that δCOP regulates Aβ production via regulating APP retrograde trafficking, and mutation of δCOP significantly decreases amyloid plaque formation while enhancing cognitive function in an AD mouse model in vivo (Bettayeb et al., 2016a; Bettayeb et al., 2016b).

Taken together, our data suggest that GSAP and its binding proteins play critical roles in regulating protein phosphorylation, trafficking, lipid metabolism, and mitochondrial function.

### GSAP directly interacts with the Fe65–APP protein complex and regulates APP phosphorylation and trafficking/partitioning

**Figure 2.**
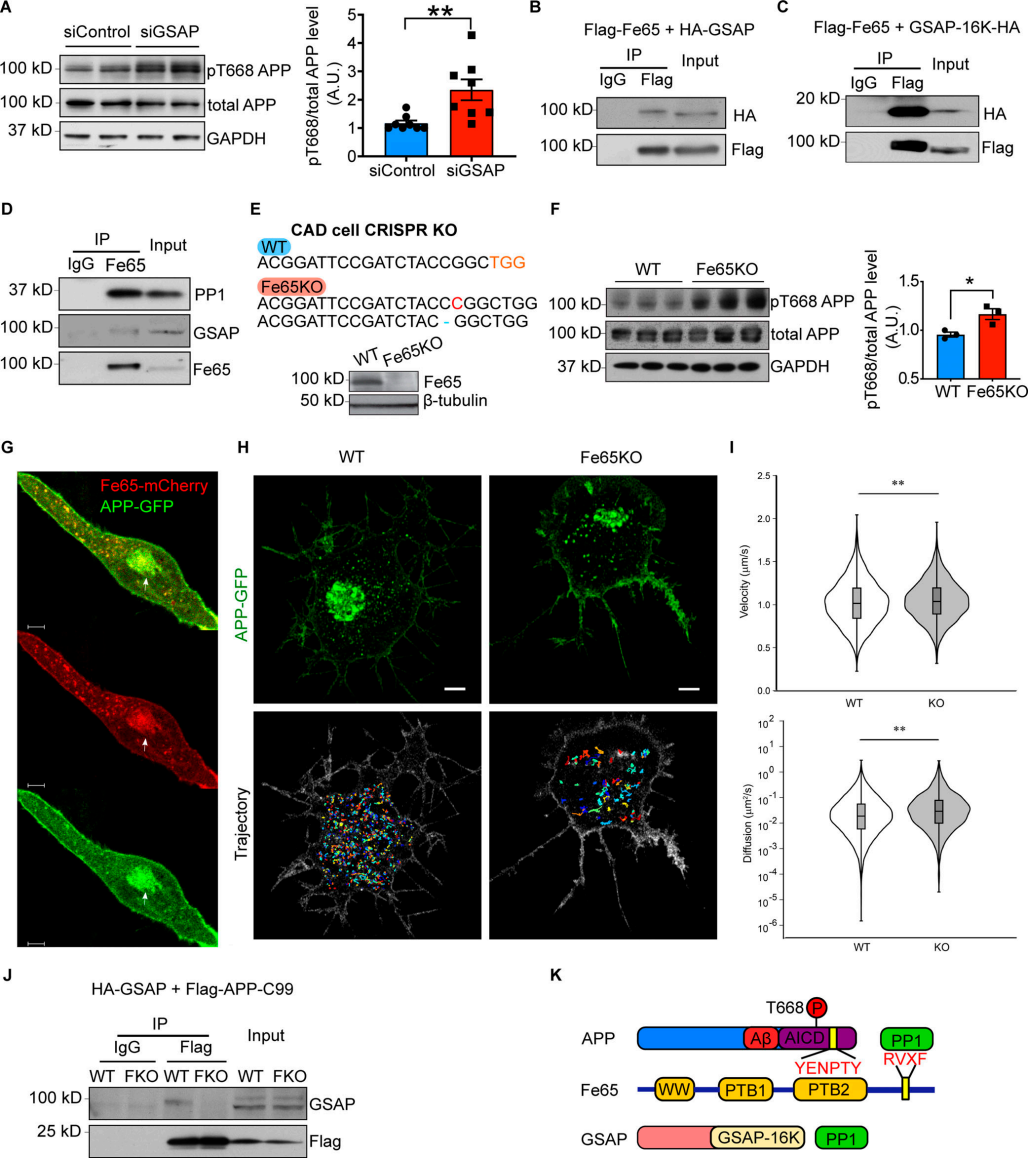
**GSAP interacts with Fe65 to regulate APP phosphorylation and trafficking. (A)** Immunoblot analysis of protein levels in N2a695 cells transfected with control or GSAP siRNA (left panel). Quantification of APP phosphorylation at Thr668 normalized to total APP level (right panel). Data represent mean ± SEM; unpaired *t* test, **, P < 0.01. pT668, phospho-Thr668. Representative data of four experiments. **(B)** Co-IP analysis of full-length GSAP (HA-tagged) interaction with full-length Fe65 (Flag-tagged) using Flag antibody in HEK293T cells. Representative data of two experiments. **(C)** Co-IP analysis of GSAP C-terminal 16K domain (HA-tagged) coprecipitation with full-length Fe65 (Flag-tagged) using a Flag antibody in HEK293T cells. Representative data of two experiments. **(D)** Co-IP analysis of endogenous Fe65 interaction with GSAP and PP1 using Fe65 antibody in HEK293T cells. GSAP was detected using an antibody from R&D Systems. Representative data of two experiments. **(E)** Genomic DNA from CAD WT and Fe65KO cells was isolated, and PCR-amplified fragments flanking the CRISPR-Cas9 cleavage site were generated. PCR fragments were cloned into TOPO vector for Sanger sequencing. A 1-bp insertion (red) and deletion (blue) was identified in Fe65KO CAD cells (upper panel). Immunoblot analysis of proteins from WT and Fe65KO CAD cells (lower panel). **(F)** Immunoblot analysis of protein levels in CAD cells transiently overexpressing APP (left panel). Quantification of APP phosphorylation at Thr668 normalized to total APP level (right panel). Data represent mean ± SEM; unpaired *t* test, *, P < 0.05. Representative data from two experiments. **(G)** Representative confocal microscopy of Fe65 (red) and APP (green) localization in differentiated CAD cells. Arrow denotes the structure of Golgi apparatus. Scale bar, 5 µm. Representative data of ten cells. A.U., arbitrary units. **(H)** Maximum intensity projection of Airyscan Z-stack of WT (top left) and Fe65KO (top right) CAD cells from 95 slices and 0.173-µm step size and generated in Imaris. Scale bars, 5 µm. The images are representative of four independent experiments. WT (bottom left) and Fe65KO (bottom right) trajectories corresponding to the representative time-lapse image series are shown in the top panel and were reconstructed in MATLAB. Trajectory minimum cutoff time is 10 s. **(I)** Violin plots showing the velocity (left) and diffusion coefficient (right) distributions of single APP-GFP vesicles in WT and Fe65KO CAD cells. The median value is shown as the horizontal line in the box. The box presents interquartile range. The distributions were compared using the Mann–Whitney *U* test (**, P < 0.001; WT V_median_ = 1.016 µm/s, KO V_median_ = 1.038 µm/s; WT D_median_ = 0.0187 μm2/s, and KO D_median_ = 0.0290 μm2/s). **(J)** Co-IP analysis of GSAP (HA-tagged) with APP-C99 (Flag-tagged) in WT and Fe65KO (FKO) CAD cells. Representative data of two experiments. **(K)** Schematic of protein domain interactions within the APP–Fe65–GSAP complex. AICD, APP intracellular domain.

Previous studies demonstrate that phosphorylation of APP at Thr668 influences APP processing via a mechanism involving its association with the lipid-raft microdomain (Matsushima et al., 2012). Moreover, our proteomic analyses revealed an enrichment of components related to protein phosphorylation in the GSAP interactome, suggesting that GSAP may modulate APP Thr668 phosphorylation. We demonstrated previously that GSAP siRNA knockdown decreases both Aβ40 and Aβ42 generation in N2a cells stably expressing human APP695 isoform (N2a695; Chang et al., 2020 *Preprint*). The GSAP siRNA significantly increased phospho-Thr668 APP levels in N2a695 cells, with no effect on total APP levels compared with control siRNA transfection (Fig. 2 A). Thr668 of APP can be phosphorylated by several kinases to regulate a variety of APP functions (Aplin et al., 1996; Iijima et al., 2000; Standen et al., 2001; Suzuki et al., 1994). In contrast, PP1 is the only protein phosphatase identified to dephosphorylate this site through the recruitment of Fe65, the well-characterized APP-binding protein (Rebelo et al., 2013). The Y2H study showed that both PP1 and Fe65 (encoded by the APBB1 gene) directly interact with GSAP (Fig. S1 C). In addition to PP1, we also confirmed the interaction of Fe65 and GSAP by co-IP assay in the cells (Fig. 2 B) and demonstrated that the GSAP-16K domain was sufficient to bind full-length Fe65 (Fig. 2 C). After GSAP antibody validation (Fig. S1 E), we further performed an endogenous co-IP experiment using a Fe65 antibody and cell lysates from human lung carcinoma cell line A549, which has high endogenous expression of both GSAP and Fe65, and showed the co-IP of endogenous Fe65 with PP1 and GSAP (Fig. 2 D). Since we observed that knockdown of GSAP decreased APP-CTF association with lipid rafts (Chang et al., 2020), these results suggest that GSAP regulates APP phosphorylation and partitioning through Fe65 interaction.

**Figure S2.**
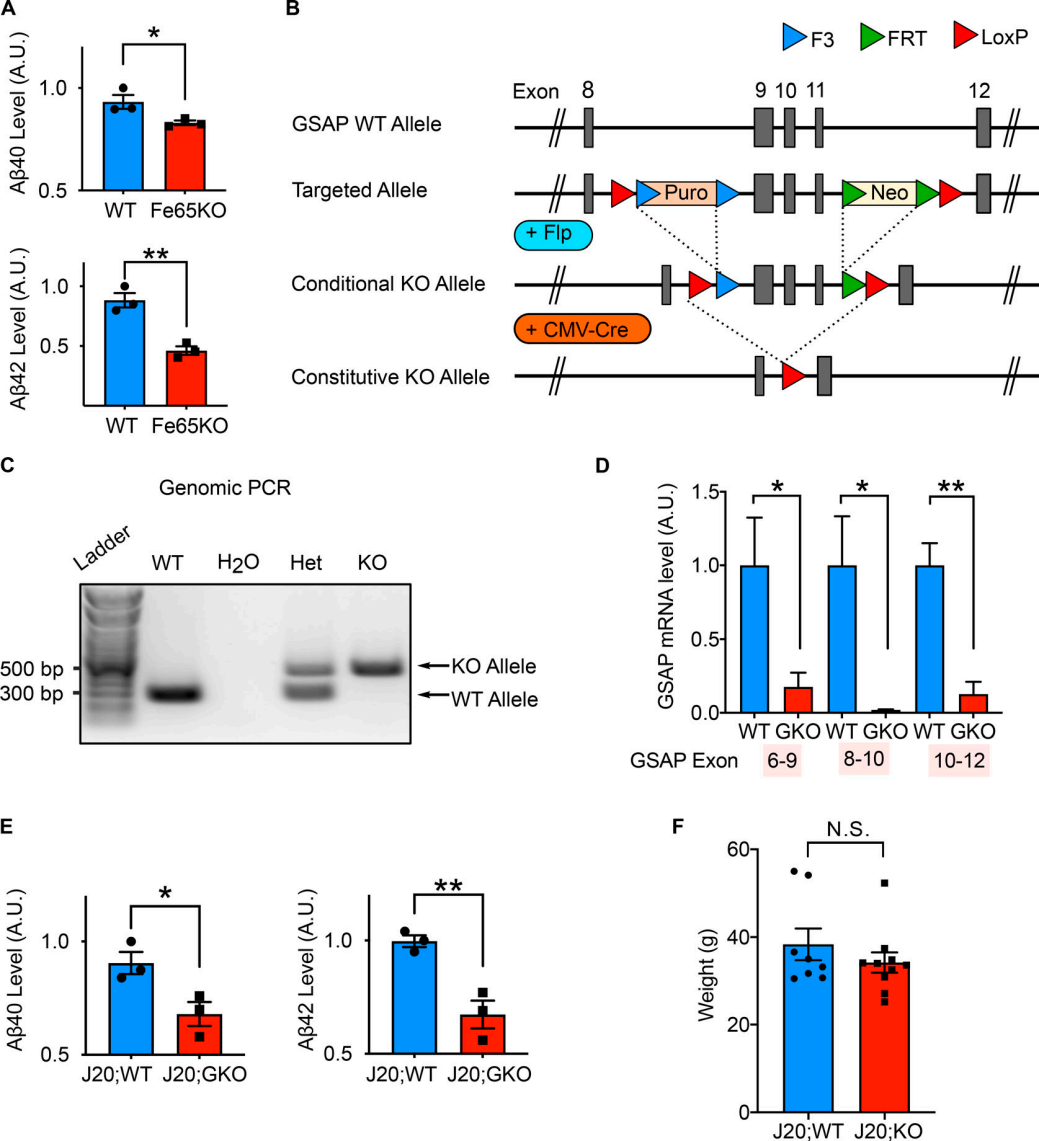
**Validation of Fe65KO CAD cells and GSAP gene deletion in mice. (A)** Quantification by ELISA of secreted Aβ40 and Aβ42 levels produced by WT and Fe65KO cells. Data represent mean ± SEM; unpaired *t* test; *, P < 0.05; **, P < 0.01. **(B)** Schematic of the gene-targeting strategy used to generate GKO mouse lines (Taconic Farms). Conditional GKO mice were crossed with CMV-Cre to generate constitutive KO mouse lines. **(C)** Genomic PCR analysis to distinguish WT (∼325 bp) and GKO (∼500 bp) alleles. Het, heterozygous of GSAP. **(D)** Quantitative PCR analysis in both WT and GKO mouse hippocampal tissues using primer sets across different GSAP exons. **(E)** Quantification by ELISA of soluble Aβ40 and Aβ42 levels in the hippocampi of J20;WT and J20;GKO mice. Data represent mean ± SEM; unpaired *t* test; *, P < 0.05; **, P < 0.01. **(F)** Weight was measured for mice used for behavioral studies at 6 mo of age. Data represent mean ± SEM; unpaired *t* test; N.S., not significant.

To further investigate potential biological effects of GSAP–Fe65 interaction, we generated Fe65 KO (Fe65KO) neuronal tumor CAD cells by CRISPR-Cas9 editing (Qi et al., 1997). Different genomic frameshifts were confirmed on both alleles of the Fe65 gene, which resulted in reduced Fe65 protein levels in Fe65KO cells (Fig. 2 E). We overexpressed APP in WT and Fe65KO CAD cells; consistent with previous observations, the phospho-Thr668 level was significantly increased in Fe65KO cells, and Aβ40 and Aβ42 levels were reduced in Fe65KO cells (Figs. 2 F and S2 A; Rebelo et al., 2013; Xie et al., 2007). Since GSAP physically interacts with Fe65 and regulates APP intracellular trafficking (Chang et al., 2020 *Preprint*), we hypothesized that Fe65 regulates APP intracellular trafficking in a manner similar to GSAP. We first characterized Fe65 and APP subcellular localization in CAD cells. Fe65 staining in differentiated CAD cells revealed Golgi-like localization in the cell body and vesicle-like localization at neurites (Fig. 2 G). In agreement with previous studies, Fe65 staining showed good overlap with APP (Sabo et al., 2003). We next determined whether Fe65 deletion could affect intracellular APP trafficking by tracking dynamics of single APP vesicles in WT and Fe65KO CAD cells. APP-GFP vesicles were tracked for 1 min under an Airyscan super-resolution microscope at 10 frames/s, and trajectories of each single APP vesicle were analyzed (Fig. 2 H). In agreement with our hypothesis, Fe65 regulated APP trafficking dynamics in a fashion similar to GSAP: Fe65KO increased APP vesicle trafficking velocity and diffusivity (Fig. 2 I). Since strong binding affinities between Fe65 and APP have been previously established (Radzimanowski et al., 2008), we hypothesized that Fe65 may be required to stabilize GSAP–APP interaction. To test this hypothesis, we compared GSAP and APP interactions in WT and Fe65KO CAD cells. HA-tagged GSAP and Flag-tagged APP-C99 (APP C terminus) proteins were coexpressed in CAD cells and subjected to immunoprecipitation (IP) using a Flag antibody. Although GSAP consistently coprecipitated with APP-C99 in WT cells, GSAP–APP complex formation was dramatically reduced in Fe65KO (FKO in the figure) CAD cells (Fig. 2 J). Taken together, this indicates that Fe65 is essential for GSAP–APP interaction and GSAP-dependent regulation of APP trafficking dynamics.

Fe65 has three well-defined protein domains (Bressler et al., 1996; Fiore et al., 1995; Guénette et al., 1996), including the PTB2 domain, which directly interacts with the APP intracellular domainin the co-crystal structure (Radzimanowski et al., 2008). The RVXF binding motif at the C terminus of Fe65 may directly interact with PP1 and recruit it to dephosphorylate APP (Rebelo et al., 2013). Together, our data suggest that GSAP is recruited by Fe65 to form a ternary APP–Fe65–PP1 protein complex (Fig. 2 K) and demonstrate that GSAP binds Fe65 directly through the GSAP-16K domain and regulates APP phosphorylation.

### GSAP regulates protein phosphorylation, trafficking, lipid metabolism, and mitochondrial function in vivo in mouse hippocampus

**Figure 3.**
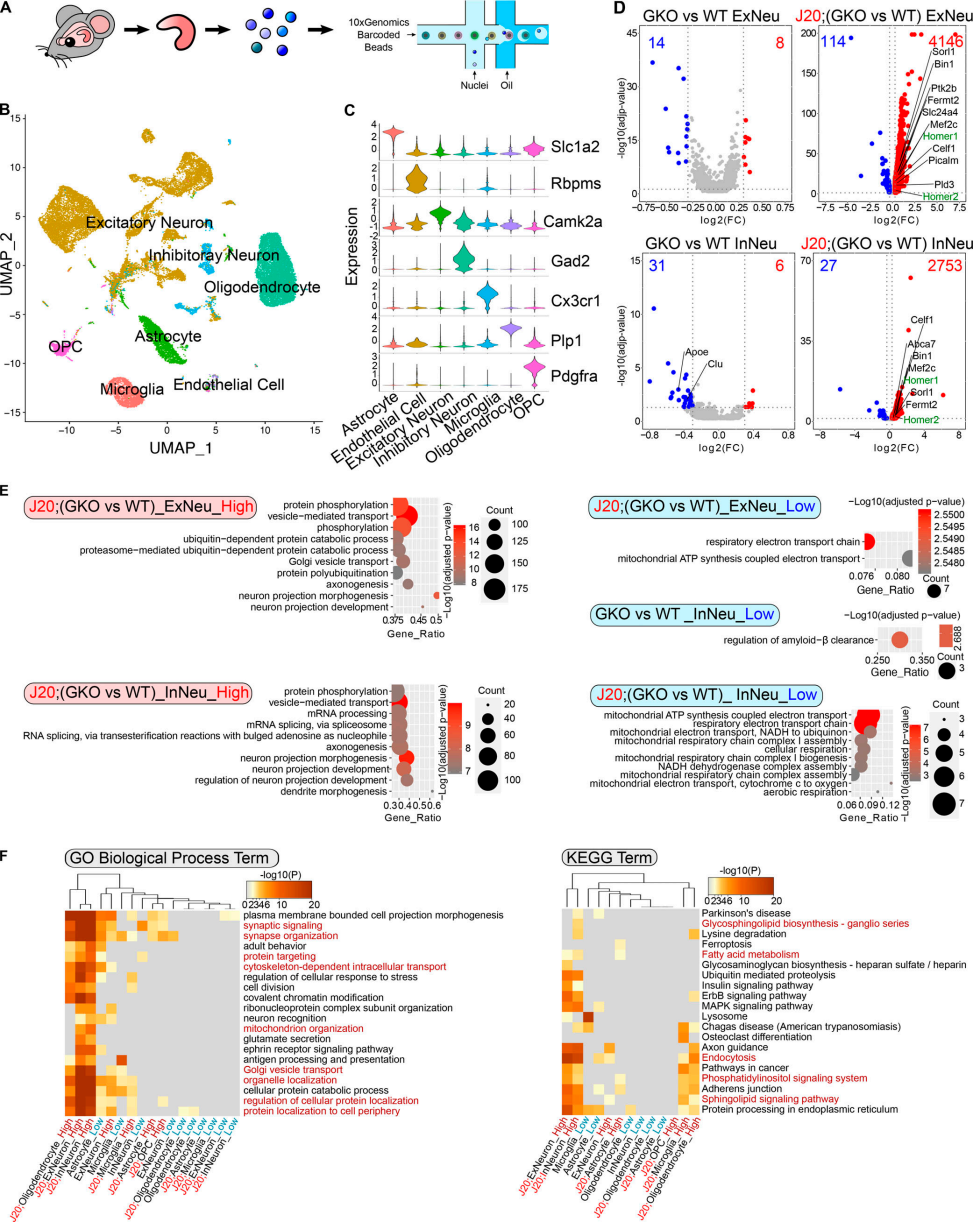
**sn-RNAseq analysis of GSAP KO mouse hippocampus. (A)** Schematic diagram of the experimental design for sn-RNAseq of mouse hippocampus (WT, GKO, J20;WT, and J20;GKO) using the 10X Genomics platform. Sequencing data from different genotypes were merged for downstream analysis. **(B)** UMAP plot showing seven major cell types clustered based on gene expression profile in an unsupervised manner. **(C)** Violin plot showing expression level of representative marker genes from different cell clusters: Slc1a2 (astrocyte; 3,220 nuclei), Rbpms (endothelial cell; 213 nuclei), Camk2a (excitatory neuron; 15,845 nuclei), Gad2 (inhibitory neuron; 1,961 nuclei), Cx3cr1 (microglia; 2,210 nuclei), Plp1 (oligodendrocyte; 7,187 nuclei), and Pdgfra (oligodendrocyte progenitor cell [OPC]; 1,287 nuclei). **(D)** Volcano plots showing DEGs in neuronal clusters comparing WT versus GKO or J20;WT versus J20:GKO. Only genes with significantly expression level change are shown (adjusted P value < 0.05; log2[fold change (FC)] < −0.3 or > 0.3). Genes with higher expression level in GKOs are highlighted in red, and genes with lower expression level in GKOs are highlighted in blue. AD risk genes are in labeled in black, whereas synaptic genes are labeled in green. **(E)** GO biological process enrichment analysis for DEGs in neuronal clusters. Top significantly changed pathways (up to 10) are shown (adjusted P value < 0.05). **(F)** Meta-enrichment analysis of common GO (left panel) and KEGG (right panel) pathways shared by both up-regulated and down-regulated DEGs from all the cell types.

**Figure S3.**
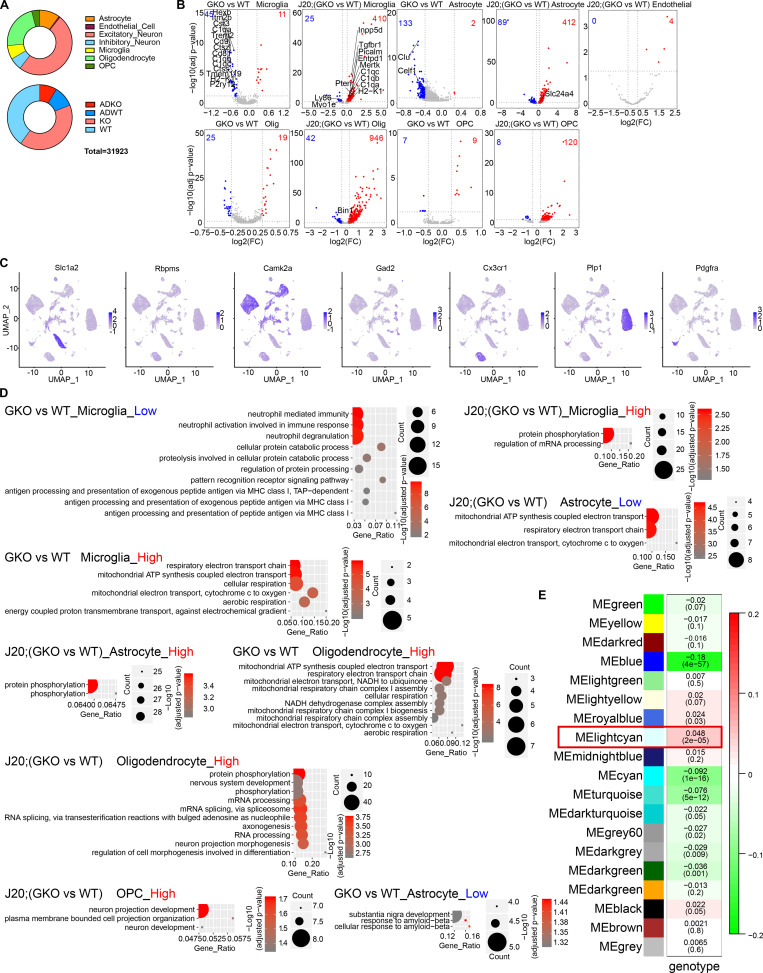
**sn-RNAseq analysis. (A)** Distribution profile of nuclei based on cell type (upper panel) or genotype (lower panel). **(B)** Volcano plot showing DEGs in different clusters in GKO versus WT or J20;GKO versus J20:WT. Only genes with significant expression level change are shown (adjusted P value <0.05; log2[fold change (FC)] < −0.3 or > 0.3). Genes with higher expression level in GKO are highlighted in red, and genes with lower expression level in GKO are highlighted in blue. **(C)** UMAP plot showing marker gene expression levels from different cell clusters. Color intensity corresponds to gene expression level. **(D)** GO biological process pathway analysis for DEGs in different cell clusters. **(E)** Module–trait relationship heatmap depicting the correlation between WGCNA gene modules and mouse genotypes. Each cell contains the corresponding correlation (top value) and P value (bottom value). ME, module eigengene.

To further investigate the pathogenic function of GSAP in disease progression, we determined effects of GSAP gene deletion in an AD mouse model. We targeted exons 9–11 of the murine GSAP gene locus by flanking loxP sites, and constitutive GKO mice were obtained by crossing GSAP conditional KO mice with a murine CMV-Cre driver line (Fig. S2 B). Genomic PCR and quantitative RT-PCR confirmed successful excision of GSAP exons 9–11 and reduced GSAP mRNA expression (Fig. S2, C and D). We next crossed GKO mice with the J20 AD mouse model (expressing human APP bearing the Swedish and Indiana mutations under the human platelet-derived growth factor beta polypeptide [PDGFB] promoter; Mucke et al., 2000) to investigate effects of GSAP deletion on AD-associated molecular and behavior changes in vivo. Consistent with previous data showing GSAP knockdown decreases Aβ levels in vivo in an AD mouse model (He et al., 2010), we observed lower Aβ40 and Aβ42 levels in the J20;GKO mouse hippocampal tissues compared with J20;WT (Fig. S2 E). GSAP expression is broadly detected in various cell types in the brain (Darmanis et al., 2015; Zhang et al., 2014; Zhang et al., 2016). To elucidate the molecular function of GSAP across various cell types in the brain, we performed sn-RNAseq on hippocampal tissues obtained from 6–7-mo-old WT, GKO, J20;WT, and J20;GKO mice (Fig. 3 A). A total of 31,923 nuclei were clustered based on their transcriptomes and visualized in uniform manifold approximation and projection (UMAP) space. Based on a previous study (Tasic et al., 2016), nuclei were annotated into seven distinct cell types in an unsupervised manner (Figs. 3 B and S3 A). The clustering results were validated by visualizing the expression level of known cell type–specific marker genes using a violin plot (Fig. 3 C). We also visualized the two-dimensional distribution of nuclei expressing these marker genes in the UMAP space (Fig. S3 C). Our data demonstrated that these marker genes were specifically enriched in the annotated cell-type clusters, confirming the accuracy of the annotation strategy.

We next sought to determine possible molecular functions of GSAP by examining differentially expressed genes (DEGs) in different cell types in the various mouse genotypes. We compared DEGs in GKO versus WT and J20;GKO versus J20;WT samples. We identified a large number of significant DEGs across cell types in GKO brain, with the exception of endothelial cells, which showed comparatively little change. The effect of GSAP deletion on DEGs was largely exacerbated in the J20 mouse model, suggesting that effects of GSAP deletion may be amplified with AD pathogenesis (Figs. 3 D and S3 B). We first compared GKO DEGs with an AD risk gene list identified from genome-wide association studies (Karch and Goate, 2015). Multiple DEGs overlapped with the AD risk gene list in different cell types (Figs. 3 D and S3 B). In both excitatory and inhibitory neurons, GSAP deletion may confer neuroprotective effects under proteotoxic AD stress, since GSAP deletion up-regulates multiple genes previously shown to reduce Aβ generation (Pld3, Sorl1, Bin1, and Fermt2). Our data also further support that loss of function in GKO-induced AD risk genes identified here may contribute to AD pathogenesis (Andersen et al., 2005; Caglayan et al., 2014; Chapuis et al., 2017; Chu et al., 2014; Cruchaga et al., 2014; He et al., 2010; Miyagawa et al., 2016; Ubelmann et al., 2017). Additionally, genes essential for synaptic function (Homer1, Homer2, and Bin1), were significantly up-regulated in both excitatory and inhibitory neurons of J20;GKO mice, suggesting that GSAP depletion may protect synaptic impairment in AD (De Rossi et al., 2020; Shiraishi-Yamaguchi and Furuichi, 2007).

We then characterized biological pathways affected by GSAP deletion. Using GO biological pathway enrichment analysis, we observed that phosphorylation and mitochondrial function were broadly altered in excitatory neurons, inhibitory neurons, oligodendrocytes, microglia, and astrocytes (Figs. 3 E and S3 D). Moreover, we observed enrichment of pathways related to vesicle-mediated transport in neurons and oligodendrocytes with GSAP deletion (Figs. 3 E and S3 D). We also performed meta-enrichment analysis using DEG lists from all the cell types to identify common biological pathways affected by GSAP depletion. Trafficking (GO term), mitochondrial function (GO term), and lipid metabolism (KEGG term) were among the top shared biological pathways in a variety of cells affected by GSAP depletion (Fig. 3 F). Notably, these biological pathways highly overlap with the GSAP functional pathways identified via proteomics, confirming the robustness of our analyses (Fig. 1, B and C).

### Characterization of GSAP function in excitatory neurons

**Figure 4.**
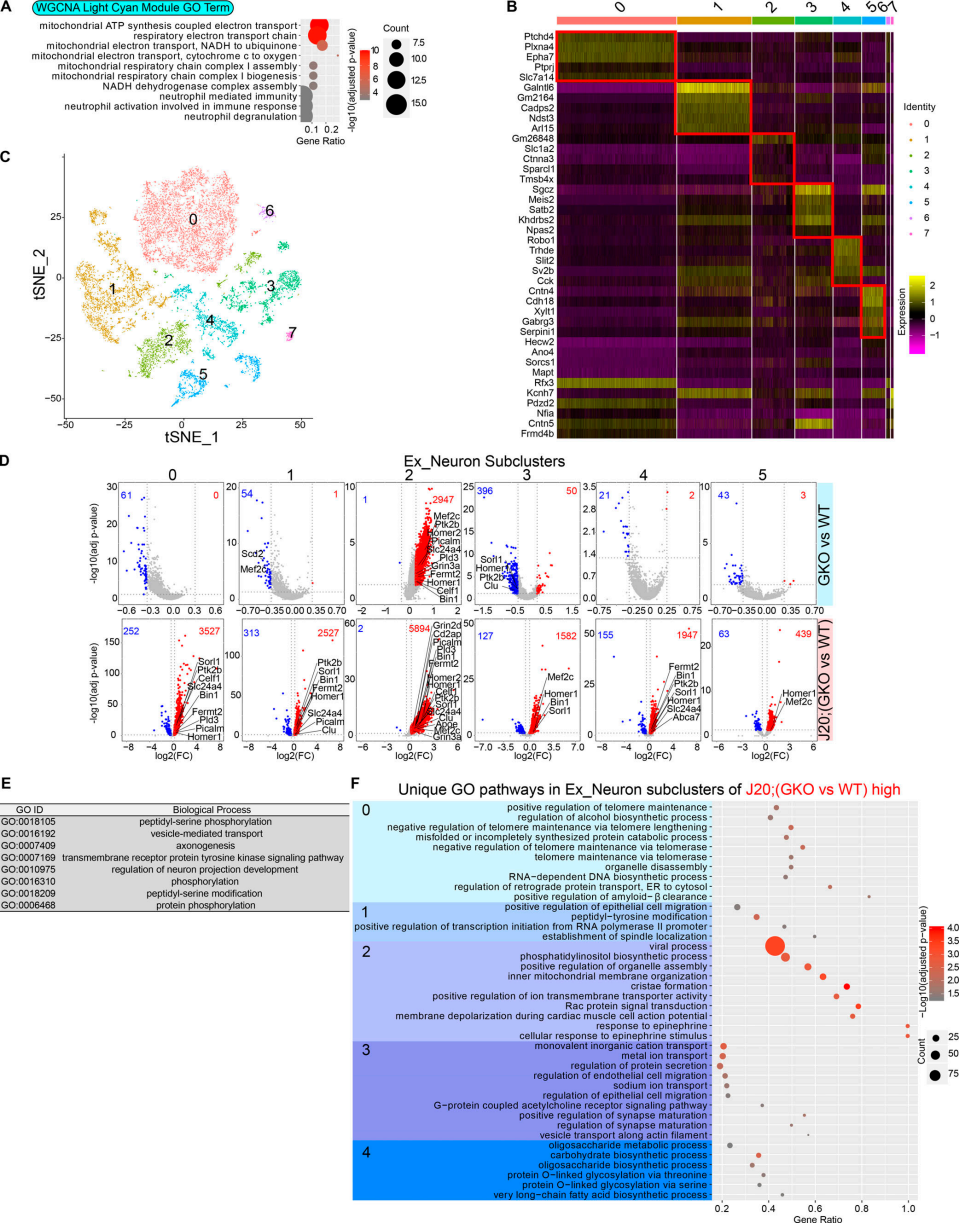
**Characterization of GSAP function in excitatory neurons. (A)** GO biological process enrichment analysis for genes enriched in the WGCNA light cyan module of excitatory neurons. Top significantly changed pathways (up to 10) are shown (adjusted P value <0.05). **(B)** Heatmap showing the expression level of the top five differentially enriched genes in each excitatory neuron subcluster. **(C)** t-Distributed stochastic neighbor embedding (t-SNE) plot depicting the excitatory neuron cluster, which is divided into eight subclusters in an unsupervised manner. **(D)** Volcano plots showing DEGs in excitatory neuron subclusters comparing GKO versus WT or J20;GKO versus J20:WT. Genes with significant changes in expression levels are shown (adjusted P value <0.05; log2[fold change(FC)] < −0.3 or > 0.3). Up-regulated genes in GKO are highlighted in red, and genes down-regulated in GKO are highlighted in blue. **(E and F)** GO biological process pathways analyses were performed with up-regulated genes comparing J20;GKO versus J20;WT mice. GO biological pathways shared by all five neuron subclusters are shown in E, and GO biological pathways uniquely overrepresented in specific clusters are shown in F.

Neurons are the major source for Aβ production (Zhao et al., 1996). Having observed that GSAP regulates Aβ production and has the highest expression level in human neurons (Darmanis et al., 2015; Zhang et al., 2016), we then focused on characterizing GSAP function in excitatory neurons, which represents the largest cell population in hippocampus. Since coexpressed genes often work in the same function cluster, we first applied weighted gene coexpression analysis (WGCNA) on DEGs from excitatory neurons to identify gene modules that function as groups (Zhang and Horvath, 2005). We determined the correlation between WGCNA gene modules and mouse genotypes and identified the light cyan WGCNA module as having the strongest correlation with genotype (Fig. S3 E). GO pathway analysis demonstrated the light-cyan module represented the mitochondrial function category (Fig. 4 A). Our data suggest that GSAP KO and/or amyloidogenesis mainly affect mitochondrial function in excitatory neurons.

Since substantial heterogeneity in gene expression was observed within excitatory neurons, we divided excitatory neurons into different subclusters. We classified eight subclusters in excitatory neurons based on nuclear transcriptomic profile (Fig. 4, B and C). Significant DEGs were identified from five of these subclusters (Fig. 4 D). Subcluster 2 from excitatory neurons exhibited the greatest effect of GSAP depletion, exemplified by the highest number of up-regulated genes even in GKO mice. These results suggest that GSAP mainly functions in the subcluster 2 excitatory neurons under physiological conditions, and this effect is potentially exacerbated in the entire excitatory neuron cluster under pathogenic conditions. Comparing J20;GKO with J20;WT, analysis of DEGs suggest that GSAP functions similarly in all five subclusters in terms of amyloidogenesis and synaptic functional genes regulation (Fig. 4 D). To elucidate functional pathway changes in the five subclusters, we searched for GO biological pathways enriched in up-regulated DEGs in J20;GKO compared with J20;WT mice and identified shared and unique functional pathways. Eight GO terms showed consistent over-representation in all five subclusters (Fig. 4 E). These results demonstrate that GSAP has a general function in the regulation of phosphorylation and trafficking across excitatory neuron subtypes. Unique pathways in individual subclusters suggest that GSAP may specifically regulate telomere lengthening in cluster 0 neurons, cell migration in cluster 1 neurons, lipid homeostasis and mitochondrial function in cluster 2 neurons, ion transport and synapse maturation in cluster 3 neurons, and oligosaccharide metabolism in cluster 4 neurons (Fig. 4 F).

### GSAP regulates lipid metabolism and mitochondrial function in the MAM

**Figure S4.**
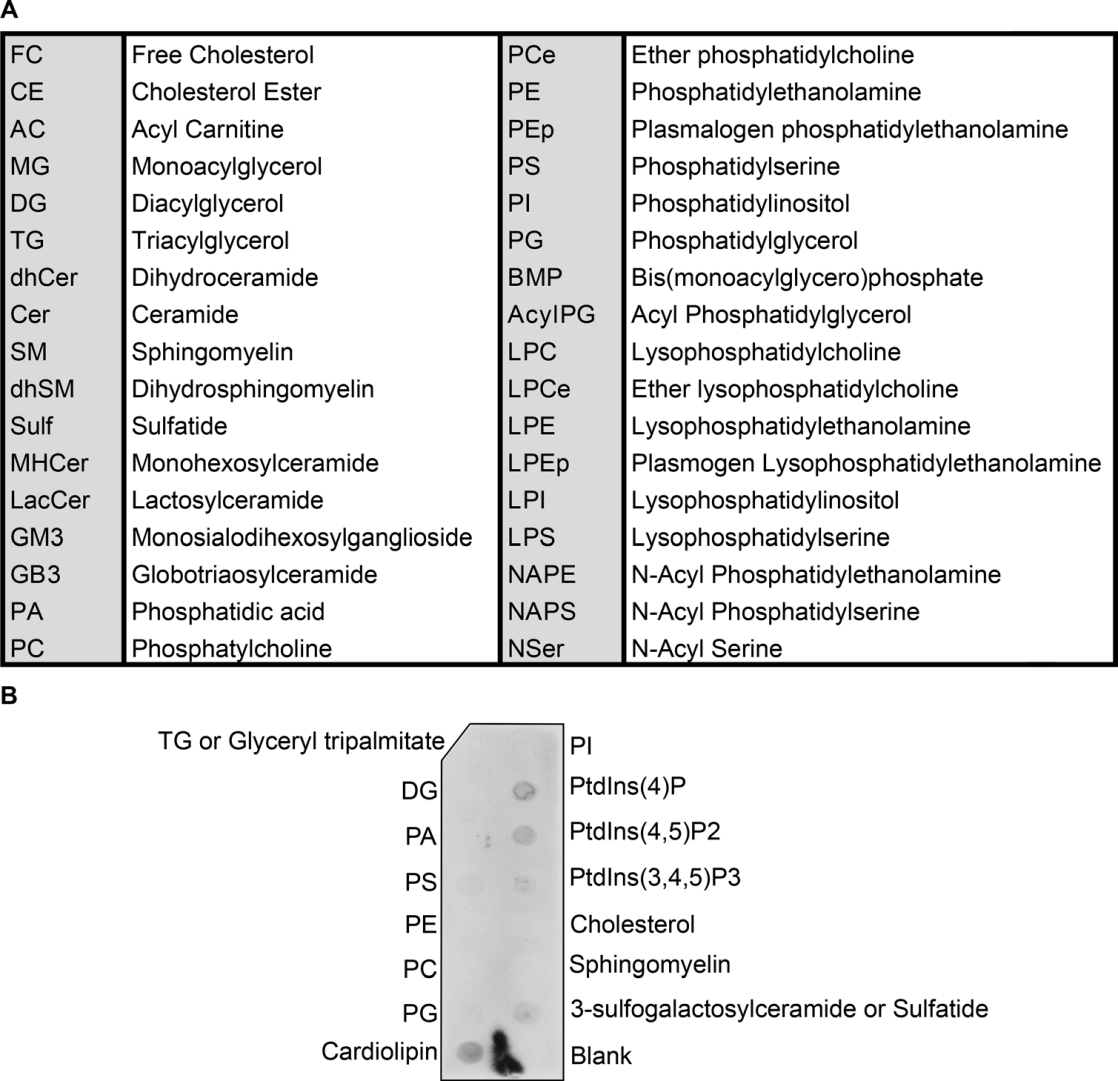
**GSAP interacts with lipids. (A)** Abbreviations used for different lipid subclasses. **(B)** Cell lysates were collected from HA-GSAP–transfected cells and overlaid onto lipid-coated membranes. HA antibody was used to detect GSAP bound to lipids.

We next sought to investigate the underlying mechanism by which GSAP regulates mitochondrial function in neuronal cells. Numerous studies have shown that the MAM is an essential hub for the regulation of lipid homeostasis, mitochondrial function, and AD pathogenesis (Area-Gomez et al., 2018; Area-Gomez et al., 2012). Specifically, APP is partitioned and processed in the MAM to generate its C99 fragment and Aβ production, which in turn have detrimental effects on lipid homeostasis and mitochondrial function (Del Prete et al., 2017; Pera et al., 2017). Multiple lines of evidence from our data suggest that GSAP may be enriched in MAM and regulate lipid homeostasis and mitochondrial function through MAM. Our proteomic and genomic data concordantly suggest that GSAP regulates trafficking, lipid metabolism, and mitochondrial function. Second, our preliminary data suggest that GSAP can interact with phospholipids and mitochondrial enriched cardiolipin (Fig. S4, A and B). GSAP interacts with the mitochondrial protein PHB, which was observed in MAM and proposed to regulate lipid homeostasis (Fig. S1 D; Osman et al., 2009; Zhang et al., 2011). Furthermore, enrichment in the MAM fraction has been reported for several GSAP-binding proteins, including APP, Psen1, Fe65, Arcn1, Copa, Copb2, Htra2, Acsl1, Hspa9, and ER lipid raft associated 2 (Erlin2; Fig. S1 A; Ma et al., 2017; Schon and Area-Gomez, 2013; Völgyi et al., 2018). Similar to GSAP, the MAM protein Erlin2 binds Psen1 and regulates γ-secretase activity toward APP processing with little or no effect on Notch (Browman et al., 2006; Teranishi et al., 2012).

**Figure 5.**
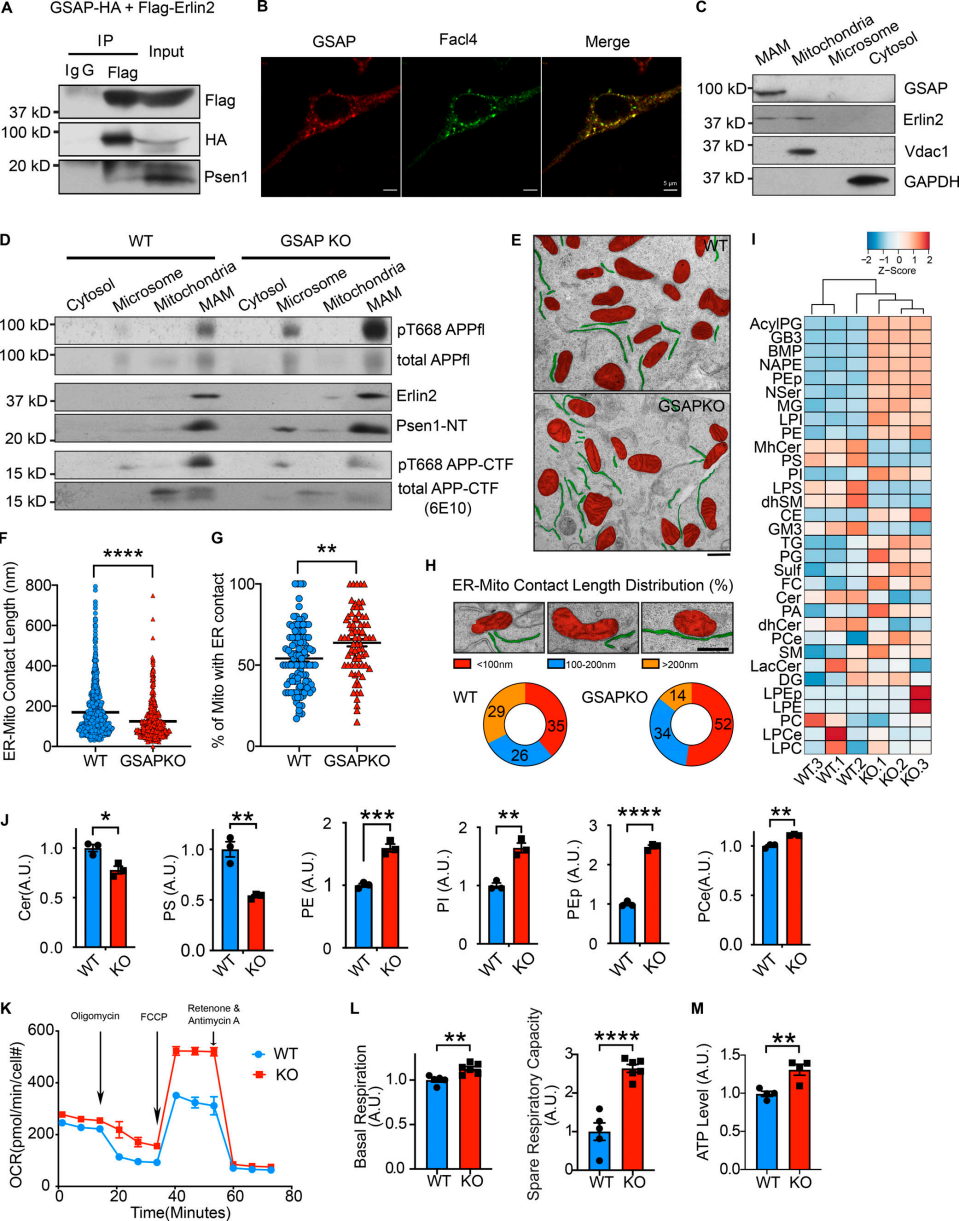
**GSAP regulates lipid metabolism and mitochondrial function through the MAM. (A)** Co-IP analysis of GSAP (HA-tagged) interaction with Erlin2 (Flag-tagged) and Psen1 using an HA antibody in N2a cells. Representative data of two experiments. **(B)** Immunofluorescence of overexpressed GSAP and MAM marker protein Facl4 in CAD cells. Scale bars, 5 µm. Representative data of five cells. **(C)** Equal amount of protein from different fractions of N2a695 cells were loaded into each lane for SDS-PAGE and immunoblot analysis with indicated antibodies. Representative data of two experiments. **(D)** Equal amounts of protein from different fractions of WT and GKO HEK293-APP cells were loaded into each lane for SDS-PAGE and immunoblot analysis. Representative data of two experiments. **(E)** Representative EM images of WT and GKO SHSY-5Y cells. Mitochondria (red) and ER (green) are highlighted. Scale bar, 500 nm. **(F)** ER–mito contact lengths are quantified on the ER side in WT (*n* = 106) and GKO cells (*n* = 76). Data represent mean ± SEM; unpaired *t* test, ****, P < 0.0001. **(G)** The proportion of mitochondria with ER contact is quantified in WT (*n* = 106) and GKO cells (*n* = 76). Images were analyzed in a blinded manner. Data represent mean ± SEM; unpaired *t* test, **, P < 0.01. **(H)** Representative EM images of three categories of ER–mito contact based on contact length (upper panel). Scale bar, 500 nm. Proportion of ER–mito contact length in each category is quantified in WT (*n* = 106) and GKO cells (*n* = 76). **(I)** Heatmap showing levels of different lipid subclasses in WT and GKO SHSY-5Y cells by lipidomic analysis. Full lipid names have been defined in Fig. S4 A. **(J)** Cer, PS, PE, PI, PEp, and ether PCe levels were quantified based on lipidomics analysis. Data represent mean ± SEM, unpaired *t* test; *, P < 0.05, **, P < 0.01; ***, P < 0.001; ****, P < 0.0001. **(K)** OCRs of WT and GKO cells were measured in real time by Seahorse assay. Data were normalized to cell count and represent mean ± SEM. Representative data of two experiments. **(L)** Basal OCR and SRC were compared between WT and GKO cells. Data represent mean ± SEM; unpaired *t* test, **, P < 0.01; ****, P < 0.0001. **(M)** Total intracellular ATP was compared between WT and GKO cells cultured in media without glucose. Data represent mean ± SEM; unpaired *t* test, **, P < 0.01.

Since we observed Erlin2 interaction with GSAP in our proteomics data, we first validated their interaction using co-IP. Flag-tagged Erlin2 pulled down HA-tagged GSAP and endogenous Psen1 (Fig. 5 A). We first directly determined if GSAP colocalizes with Facl4, a known MAM marker (Area-Gomez et al., 2009). In CAD cells, GSAP staining showed high colocalization with Facl4 staining in an immunofluorescence assay (Fig. 5 B). Moreover, we tested whether GSAP is enriched in MAM by cell fractionation (Lewis et al., 2016). To distinguish subcellular fractions, we first analyzed proteins previously shown to be located in different cellular compartments. Consistent with previous studies, we observed Erlin2 in the MAM fraction, Vdac1 in the mitochondria fraction, and GAPDH in the cytosolic fraction (Fig. 5 C). In the same assay, we found that GSAP was enriched in MAM (Fig. 5 C). Using the cell fractionation assay, we investigated the distribution of APP in the MAM with previously generated WT and GSAP KO HEK293-APP cells (Wong et al., 2019). GSAP KO did not affect Erlin2 or Psen1 enrichment in MAM (Fig. 5 D). Consistent with our GSAP RNAi data, Thr668 phosphorylation of full-length APP was increased in MAM of GKO cells (Fig. 5 D). However, in MAM of GKO cells, Thr668 phosphorylation of APP-CTF and total APP-CTF were decreased, consistent with our data showing GSAP knockdown decreases APP-CTF association with lipid rafts (Chang et al., 2020 *Preprint*). As suggested previously, APP-CTF distribution in MAM may be regulated by its Thr668 phosphorylation (Matsushima et al., 2012). It is possible that Thr668 phosphorylation of APP recruits the proline cis-trans isomerase Pin1, which affects APP-CTF distribution to MAM by changing the protein conformation (Pastorino et al., 2006). Next, we analyzed ER–mitochondria (ER–mito) association directly using electron microscopy (EM). We observed close ER–mito contacts in both WT and previously established GKO SHSY-5Y cells (Fig. 5 E; Wong et al., 2019). Quantification of ER–mito contacts demonstrated that ER–mito contact length significantly decreased in GKO cells (Fig. 5 F), which correlates with decreased MAM function (Area-Gomez et al., 2012). We also observed an increase proportion of mitochondria with ER contact in GKO cells (Fig. 5 G). Further studies are needed to correlate this phenotype with MAM and/or mitochondria function. Specifically, a majority of the ER–mito contacts were very short in GKO cells (<100 nm), suggesting GSAP is an essential regulator for ER–mito interaction (Fig. 5 H).

To evaluate if GSAP regulates lipid homeostasis, we systematically assessed changes of different lipid classes via lipidomic analysis in WT and GKO SHSY-5Y cells. We noticed significant changes in different classes of lipid levels after GSAP KO (Figs. 5 I and S4 A). Similar to γ-secretase inhibition (Area-Gomez et al., 2012), GSAP KO increased phosphatidylethanolamine (PE), confirming effects of GSAP deletion on MAM function (Fig. 5 J). Notably, we also observed that GSAP KO decreased cellular ceramide (Cer) and phosphatidylserine (PS) levels, and increased phosphatidylinositol (PI), plasmalogen PE (PEp), and ether phosphatidylcholine (PCe) levels (Figs. 5 H and S4 A). The cellular lipid profile changes showed the opposite direction of AD pathogenesis (Kosicek and Hecimovic, 2013), suggesting that GSAP KO may reverse the cellular lipid environment that facilitates AD pathogenesis.

Amyloidogenic processing of APP in MAM, the intracellular lipid raft–like domain, has been demonstrated to directly regulate lipid homeostasis and mitochondrial function. Specifically, MAM accumulation of APP-C99 triggers the up-regulation of Cer level, which leads to mitochondrial oxidative phosphorylation defects in AD (Area-Gomez et al., 2018; Area-Gomez et al., 2019). Interestingly, we have observed that GSAP is enriched in lipid-raft microdomains, and knockdown of GSAP decreases APP-CTF association with lipid rafts (Chang et al., 2020 *Preprint*). Importantly, Cer level was significantly decreased after GSAP KO (Fig. 5 J). We therefore tested if GSAP KO affected mitochondria bioenergetic capacity. We measured mitochondrial oxygen consumption rate (OCR) in WT and GSAP KO cells using the Seahorse assay (Fig. 5 K). Compared with WT, GSAP KO significantly increased both basal respiration and spare respiratory capacity (SRC; Fig. 5, K and L), which is critical for neuronal survival under cellular stress (Desler et al., 2012). Consistently, total ATP levels were increased in GSAP KO cells compared with WT (Fig. 5 M). Our results suggest that GSAP deficiency improves mitochondrial function, which showed deficits early in AD pathogenesis (Terada et al., 2020; Yao et al., 2009). In summary, our data demonstrate that GSAP is enriched in the MAM and regulates lipid homeostasis and mitochondrial function in the MAM.

### Knockout of GSAP rescues novel object recognition behavior in the J20 AD mouse model

**Figure 6.**
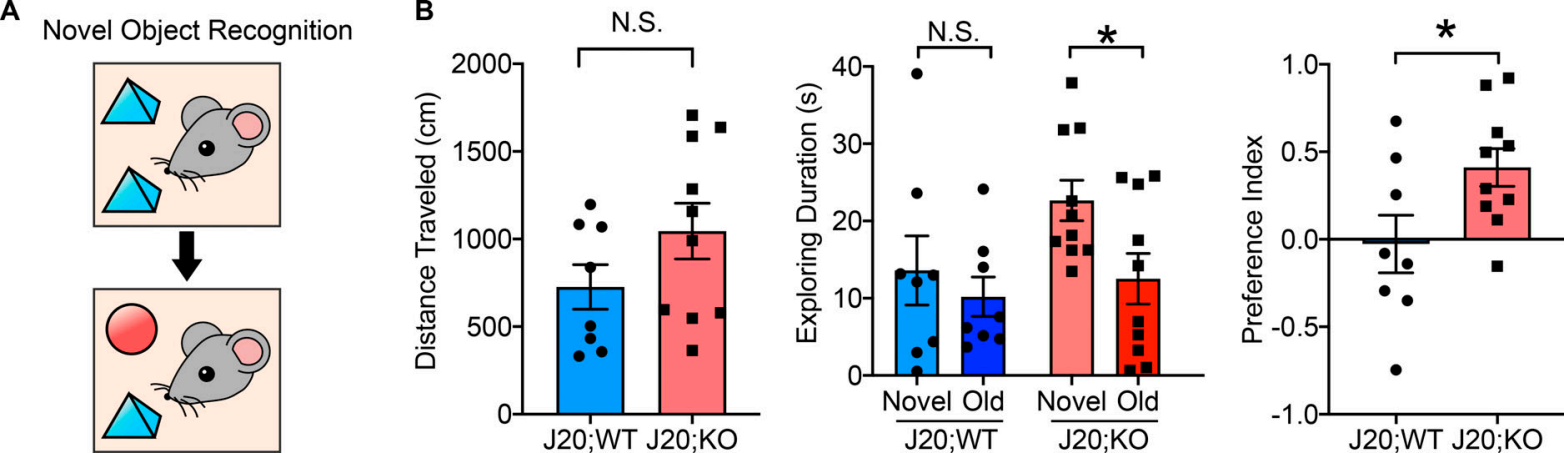
**GKO rescues novel object recognition behavior in the J20 mouse model. (A)** Diagram representing the novel object recognition test. **(B)** Memory behavior in 6-mo-old J20;WT mice (five males and three females) and J20;GSAP KO mice (J20;KO; four males and six females) were evaluated by novel object recognition test. Left: Total distance traveled during the rehabituation phase was quantified. Middle: Exploration times for old and novel objects during the choice phase were quantified in both genotypes. Right: Preference index = (time novel − time familiar)/(time novel + time familiar). Data represent mean ± SEM; unpaired *t* test; *, P < 0.05; N.S., not significant.

To further investigate the biological function of GSAP, we assessed whether GSAP impacts cognitive function in an AD mouse model by examining alterations in mouse behavior in J20;GKO mice. Since J20 mice exhibit major cognitive deficits at 5–7 mo of age, we used 6-mo-old J20;WT and J20;GSAP^−/−^ (J20;KO) mice for behavioral analysis (Harris et al., 2010). We did not observe weight differences at 6 mo of age (Fig. S2 F). Novel object recognition tests were used to evaluate whether GSAP affects recognition memory in AD mice (Fig. 6 A). During the rehabituation phase, no difference in travel distance was observed, indicating comparable locomotor activity with GSAP deletion (Fig. 6 B, left panel). During the choice phase, J20;WT mice spent a similar amount of time exploring novel and old objects, whereas J20;KO mice spent significantly more time with the novel object (Fig. 6 B, middle panel). Preference index showed that J20;GKO mice had significantly greater preference toward the novel object (Fig. 6 B, right panel). These results indicate that GSAP KO restores the recognition memory deficits in the J20 AD mouse model (Mucke et al., 2000).

### Transcriptional regulation of GSAP correlates with human aging and AD

**Figure 7.**
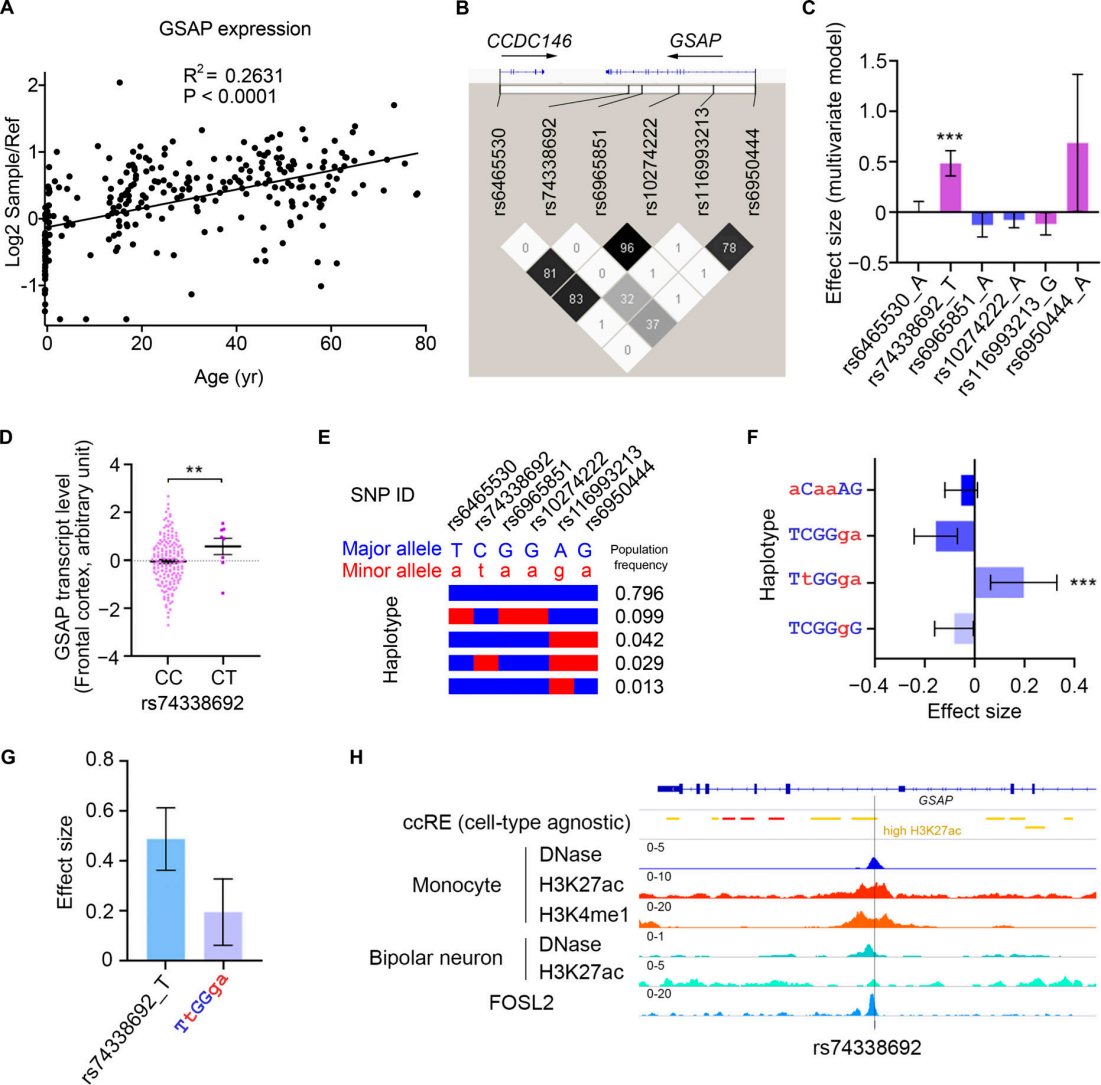
**GSAP is involved in the pathogenesis of AD. (A)** Human GSAP transcript levels are up-regulated with age. Data were obtained from BrainCloud. **(B)** Linkage disequilibrium analysis of GSAP AD risk variants that are located in cis-regulatory elements. The color map corresponds to the pairwise R^2^ values between variants, with values of R^2^ also marked in the plot. **(C)** Association between GSAP AD risk variants and brain GSAP transcript level. The plot shows the effect sizes and corresponding standard errors obtained from the meta-analysis of the results from different brain regions. Data represent effect size ± SE. ***, P < 0.001. **(D)** Association between rs74338692 and GSAP transcript level in the frontal cortex (*n* = 167 and 8 for CC and CT, respectively). Data represent mean ± SEM. **, P < 0.01. **(E)** Haplotype structure defined by the GSAP AD risk variants. Each bar represents a haplotype defined by the minor (red) or major (blue) alleles of six selected variants, with the population frequency (European population from 1000 Genomes phase 3 data; *n* = 503) marked on the right side of corresponding haplotypes. **(F)** Association between GSAP haplotypes and brain GSAP transcript levels. The uppercase (blue) and lowercase (red) letters denote the major and minor alleles of the corresponding variants, respectively. The plot shows the effect sizes and corresponding standard errors obtained from the meta-analysis of the results from different brain regions. Data represent effect size ± SE. ***, P < 0.001. **(G)** Comparison between rs74338692- and rs74338692-associaited haplotypes for their associations with brain GSAP transcript levels. The plot shows the effect sizes and corresponding SEs obtained from the meta-analysis of the results from different brain regions. Data represent effect size ± SE. **(H)** Database evidence suggest the potential regulatory roles of rs74338692. From top to bottom: ccRE, cell type–agnostic annotation for cis-regulatory elements from SCREEN database (hg19 version); yellow, cis-regulatory regions with high H3K27ac signal; DNase, the normalized signal of DNase I–hypersensitive sites sequencing data in different cell types; H3K4me1, the normalized signal of H3K4me1 ChIP-seq data in monocytes; H3K27ac, the normalized signal of H3K27ac ChIP-seq data in different cell types; FOSL2, the normalized signal of FOSL2 ChIP-seq in HepG2 cells. The heights of each track were labeled on the upper-left corner of corresponding tracks, with cell-type information labeled on the left of the tracks.

**Figure S5.**
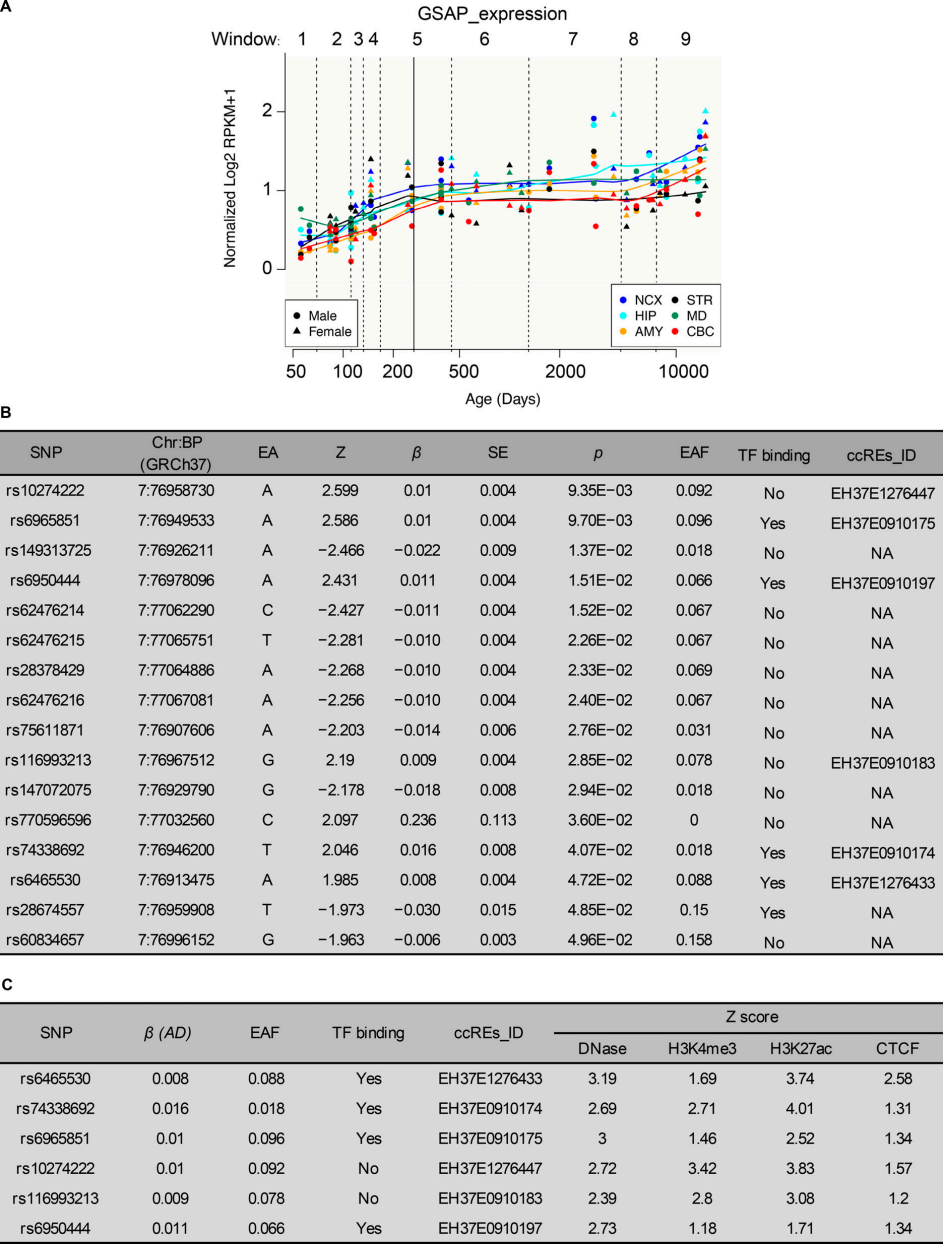
**SNPs affecting GSAP expression in human brain tissues. (A)** Human GSAP transcript levels are up-regulated with age in different brain regions. Data were obtained from PsychENCODE. RPKM, reads per kilobase of transcript per million reads mapped; NCX, neocortex; HIP, hippocampus; AMY, amygdala; STR, striatum; MD, mediodorsal nucleus of thalamus; CBC, cerebellar cortex. **(B)** Variants located in the GSAP locus identified from a European-descent population with significant association with AD (P < 0.05). Data were retrieved from the meta-analysis results from Jansen et al. (2019) and ranked by P value. β, effect size; BP, base pair; ccREs, candidate cis-regulatory elements; Chr, chromosome; EA, effective allele; NA, not available; EAF, effective allele frequency; TF, transcription factor. **(C)** AD-associated GSAP variants resided in the candidate cis-regulatory elements as reported by the ENCODE SCREEN database (hg19) listed by genomic coordinates.

Since aging is the highest risk factor for AD, we first analyzed GSAP mRNA expression across different human brain regions at different ages. Data from both BrainCloud (Colantuoni et al., 2011) and PsychENCODE (http://www.psychencode.org) independently provided evidence that GSAP transcripts increased with age across varying human brain regions (Figs. 7 A and S5 A). Increased GSAP mRNA expression with age supports previous findings that GSAP levels are significantly elevated in AD patient brain with severe pathology and cognitive deficits (Perez et al., 2017; Satoh et al., 2011; Zhu et al., 2014). These results indicate GSAP may contribute to human AD during aging.

Multiple GSAP SNPs have been previously associated with AD (Floudas et al., 2014; Zhu et al., 2014). A candidate genetic study has identified the potential association between GSAP promoter variants and AD risk in a Chinese AD cohort (Zhu et al., 2014). To conduct a comprehensive analysis of AD genetic risk for GSAP, we investigated the results from the up-to-date AD meta-analysis (Jansen et al., 2019). By querying the summary statistics obtained from the AD meta-analysis generated from ∼450,000 individuals (*n* = 455,258), several noncoding variants (P < 0.05) resided in the GSAP locus that exerted AD risk were identified (Figs. 7 B and S5 B). Six of the identified variants were located in the annotated cis-regulatory elements, with the majority of them being found in the regions with transcription factor–binding events (Fig. S5 C). To investigate their possible effects on GSAP level, association analysis was conducted between the identified variants and the brain GSAP transcript level, with only rs74338692 exerting significant association after meta-analysis summarizing results from 13 brain regions (P < 0.001; Fig. 7 C; see Fig. 7 D for a demonstration of the association between rs74338692 and GSAP expression in the frontal cortex).

As rs74338692 is in moderate linkage disequilibrium with other SNPs residing in the regulatory regions (R^2^ > 0.3), the observed regulatory effects of rs74338692 on brain GSAP levels may also be contributed by other regulatory SNPs that cosegregate with rs74338692. Haplotype analysis regarding the six SNPs was conducted, and there was only one haplotype (TtGGga) harboring the AD risk allele rs74338692, which also harbored the risk alleles of two other regulatory SNPs (Fig. 7 E). Further association analysis again revealed that only this haplotype, TtGGga, was significantly associated with brain GSAP transcript levels (P < 0.001; Fig. 7 F). Notably, this haplotype, although harboring three risk alleles, did not exert a higher effect size for modulating GSAP brain transcript level as compared with the rs74338692 alone (Fig. 7 G). Thus, the observed regulatory effect of rs74338692 was not contributed by other regulatory SNPs, and rs74338692 might be a major genetic factor that modulates the expression of GSAP in the brain. Subsequent investigation of epigenetic profiles in rs74338692-associated genomic region revealed its overlap with the high H3K27ac signal, a marker for enhancer activity, in a cell type–agnostic manner. By specifically examining the epigenomic profiles of monocytes (with high GSAP expression), additional signals for regulatory regions or enhancer activity, including DNase, H3K4me1, and H3K27ac, were again observed in the rs74338692-associated genomic region. Notably, this region also exerted regulatory property in neuronal cell types, including DNase and H3K27ac. Moreover, transcription factor–binding activity was also observed in this region, as suggested by the chromatin immunoprecipitation sequencing (ChIP-seq) results of FOSL2, a subunit of the AP-1 transcription factor complex (Fig. 7 H). In summary, our results suggest a potential regulatory function of the rs74338692-harbored genomic region, which might be the underlying mechanism of how the AD risk GSAP variant rs74338692 may lead to elevated GSAP levels in the brain.

## Discussion

Clinical AD trials have so far tested two γ-secretase inhibitors (semagacestat and avagacestat), the failure of which highlights the importance of developing selective γ-secretase modulators (Coric et al., 2012; Doody et al., 2013; Karran and Hardy, 2014; Mekala et al., 2020; Nie et al., 2020). We previously implicate GSAP as an attractive target for selective γ-secretase modulation based on two related mechanisms: (1) GSAP specifically regulates γ-secretase catalytic activity toward APP by modulating PS1 conformation; and (2) GSAP regulates APP trafficking and partitioning into lipid-raft microdomain, where γ-secretase is enriched (Chang et al., 2020 *Preprint*; He et al., 2010; Wong et al., 2019). Using proteomics and sn-RNAseq, our current work unbiasedly uncovered potential molecular functions of GSAP in the regulation of protein phosphorylation, trafficking, lipid metabolism, and mitochondrial function. Many of these pathways are directly affected by γ-secretase and dysregulated in late-onset AD, further supporting that selective γ-secretase modulation via GSAP could be beneficial in AD treatment.

GSAP is broadly expressed in various brain cell types and shows highest expression in neurons in humans (Darmanis et al., 2015; Zhang et al., 2014; Zhang et al., 2016). GSAP deletion significantly changes transcriptomic profiles in almost all cell types, including neurons. Pathway analysis of our proteomic and genomic data concordantly revealed protein phosphorylation and trafficking as the top GSAP-regulated biological pathways shared by different cell types. Previous studies have extensively shown that neuronal APP trafficking is regulated by protein phosphorylation and represents one of the most essential pathways in AD pathogenesis (Haass et al., 2012). Recently, we observed that APP trafficking and partitioning in neuronal cells is regulated by GSAP (Chang et al., 2020 *Preprint*), which may occur through the novel GSAP–Fe65–APP–PP1 protein complex described here. Since conflicting results have been reported with respect to the direct interaction of GSAP and APP, our current data favor a molecular model where Fe65 recruits GSAP–PP1 to dephosphorylate APP and regulate its trafficking and partitioning to lipid rafts (Angira et al., 2019; Deatherage et al., 2012; Savolainen et al., 2015). Although it has been demonstrated that Thr668 phosphorylation of APP diminishes its interaction with Fe65 (Ando et al., 2001), it remains unclear how APP phosphorylation regulates possible multiple intermolecular interactions within the large GSAP–Fe65–APP–PP1 protein complex, which will require substantial future exploration. Additionally, our sn-RNAseq data also indicate that GSAP may regulate microglia activation, which needs further exploration.

Lipid metabolism and mitochondrial function are the top biological pathways regulated by GSAP. It has been established that mitochondria dysfunction contributes to AD pathogenesis (Guo et al., 2020; Wang et al., 2020). Notably, mitochondrial function had the strongest correlation with GSAP KO and/or amyloidogenesis in excitatory neurons. Previous work suggested that the MAM is a central hub for lipid metabolism and mitochondrial function regulation (Area-Gomez et al., 2018). It was demonstrated that the amyloidogenic processing of APP in the MAM is responsible for the dysregulation of lipid metabolism (Del Prete et al., 2017; Pera et al., 2017). Interestingly, we demonstrated that GSAP is localized in the MAM, the intracellular lipid raft–like domain, and knockdown of GSAP decreases APP-CTF accumulation in lipid rafts and decreases Aβ production. Hence GSAP may function through modulating both APP partitioning to the MAM and γ-secretase activity in the MAM to regulate lipid metabolism and mitochondrial function. Indeed, GSAP depletion decreases ER–mito contacts, which were shown to be increased in different models of AD pathogenesis (Area-Gomez et al., 2012; Del Prete et al., 2017; Hedskog et al., 2013; Martino Adami et al., 2019). Notably, GSAP depletion significantly decreases the Cer level, a known apoptogenic mediator and important neurodegeneration regulator, which is commonly increased in human AD brain (Jana et al., 2009; Kolesnick and Krönke, 1998; Kosicek and Hecimovic, 2013). Cellular Cer level can be regulated by various amyloidogenic products of APP: APP-C99 accumulation in the MAM increases Cer synthesis (Pera et al., 2017), while different forms of Aβ also induce Cer synthesis and cell death in neurons and glial cells (Ayasolla et al., 2004; Lee et al., 2004; Malaplate-Armand et al., 2006; Zeng et al., 2005). In addition to Cer, GSAP depletion reverses the cellular lipid environment in the opposite direction of AD pathogenesis. Depletion of GSAP increases PE, PI, PEp, and PCe levels and decreases PS levels. Human AD brain showed consistent decreases in PE, PI, and PEp compared with control (Kosicek and Hecimovic, 2013). Moreover, PCe was decreased in the PS1/APP AD mouse model in the cortex compared with control (Ojo et al., 2019), and PS can mediate synaptic pruning by microglia as an “eat-me” signal (Scott-Hewitt et al., 2020). It was also demonstrated that PEp can reduce γ-secretase activity for Aβ production, preventing neuronal death (Su et al., 2019). We also observed that GSAP KO also decreases the level of lysophosphatidylserine, the up-regulation of which can promote neurodegeneration through microglia (Blankman et al., 2013).

Lipid metabolism significantly contributes to mitochondrial function. Previous work has demonstrated that the increase in cellular Cer may be the major cause of subsequent mitochondrial dysfunction (Pera et al., 2017). Moreover, PE deficiency can also impair mitochondrial function and morphology (Tasseva et al., 2013). In agreement with this idea, the lipid profile changes after GSAP depletion may largely contribute to the improvement of mitochondrial function. It is of particular interest that GSAP depletion significantly increases mitochondrial SRC. SRC is thought to generate extra energy supply to maintain cellular function, especially under stress (Sansbury et al., 2011). Consistently, enhanced SRC promotes cell survival, whereas reduced SRC may contribute to cell death (Nickens et al., 2013; Yadava and Nicholls, 2007). In AD, deficiency of SRC was shown to contribute to neuropsychological changes (Bell et al., 2020). Since mitochondrial function is one of the early deficits in AD (Terada et al., 2020; Yao et al., 2009), reduction of GSAP level may delay the pathogenesis of AD. Since we identified that GSAP interacts with cardiolipin, the important lipid in maintaining mitochondrial inner membrane integrity and regulating system uncoupling (Koshkin and Greenberg, 2002; Nguyen et al., 2016), the increase of SRC may be explained by GSAP’s role in uncoupling proton flow to ATP synthesis. Lastly, GSAP also interacts with several components of the ER-associated degradation machinery, a protein quality-control mechanism that regulates mitochondrial function through MAM and is critical in AD pathogenesis (Zhou et al., 2020; Zhu et al., 2017). Further studies will be needed to characterize the functional interactions between ER-associated degradation and GSAP in AD pathogenesis.

Similar to IFITM3, the newly identified γ-secretase modulatory protein (Hur et al., 2020), GSAP level is significantly induced by inflammatory responses and up-regulated by aging and AD pathogenesis in humans. Its expression is induced by LPS and IFNγ in macrophages (Orecchioni et al., 2019) and LPS alone in primary microglia cells (He et al., 2018). Our results indicate that the AD risk GSAP variant elevates its brain transcript level and may play an important role in the pathogenesis of AD. It would be interesting to further investigate the overexpression effects of GSAP in the most disease-relevant cell types, namely human neurons and microglia cells derived from stem cell/fibroblast. Furthermore, the GSAP homologue in *Drosophila* was found to genetically interact with the intermediate early transcription factor AP-1 and consequently regulate neuronal AP-1 function (Franciscovich et al., 2008). Since AP-1 function is critical for neuroplasticity, learning, and memory, it would be interesting to investigate the interaction of GSAP with AP-1 in mammalian system and further determine its function in learning and memory (Gallo et al., 2018).

**Figure 8.**
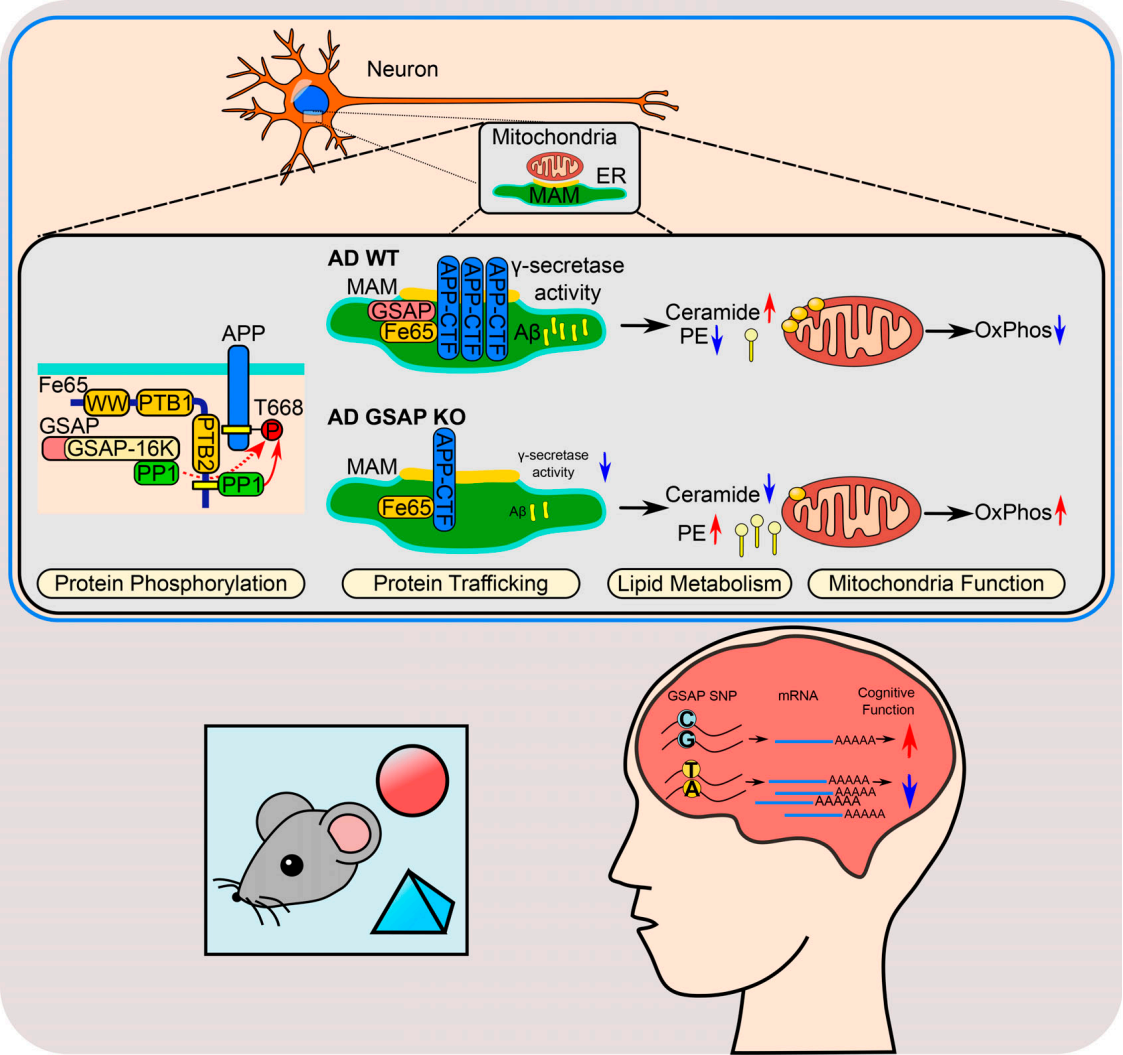
**Summary model.** GSAP is involved in late-onset AD–related pathways, including protein phosphorylation, trafficking, lipid metabolism, and mitochondrial function. In neurons, GSAP forms a complex with Fe65–PP1–APP to regulate APP phosphorylation; depletion of GSAP decreases APP-CTF partitioning into lipid rafts (MAM) as well as γ-secretase activity for Aβ generation. These amyloidogenic products have detrimental effects on the cellular lipid homeostasis. Depletion of GSAP maintains a lipid environment with up-regulated PE and down-regulated Cer, which improves mitochondrial function. Functionally, we discovered that GSAP deletion restored novel recognitive function in an AD mouse model and provided evidence that a GSAP SNP is associated elevated GSAP expression correlated with AD. OxPhos, oxidative phosphorylation.

In summary, our work indicates that GSAP regulates lipid metabolism and mitochondrial function in the MAM by modulating both APP partitioning and γ-secretase catalytic activity, suggesting GSAP is a pathogenic component of human AD and exacerbates AD phenotypes in AD mice. Thus, reducing GSAP levels may ameliorate cognitive deficits in AD (Fig. 8).

## Materials and methods

### Mouse strains

All animal experiments were approved by The Rockefeller University Institutional Animal Care and Use Committee. Mice were maintained in a C57BL/6N genetic background and housed in rooms on a 12-h dark/light cycle interval with food and water available ad libitum. GSAP conditional KO mice were constructed at Taconic Farms by targeting exons 9–11 of GSAP. Constitutive GKO mice were generated by crossing GSAP conditional KO mice with CMV-Cre mice. J20 mice were purchased from The Jackson Laboratory (B6.Cg-Zbtb20Tg(PDGFB-APPSwInd)20Lms/2Mmjax; Mutant Mouse Resource and Research Center stock no. 34836-JAX). Both male and female littermates obtained from in vitro fertilization were used for behavioral tests and sn-RNAseq.

### Novel object recognition test

During the habituation phase, mice were placed in an empty open arena for 10 min. 24 h later, mice were placed into the empty arena again for 5 min for the rehabituation phase. Subsequently, two identical objects were fixed to the floor in two corners of the box, and mice were allowed to explore for 10 min. 24 h later, one familiar object was replaced by a novel object, and mice were allowed to explore for 10 min for the choice phase. Mice interacting with an object for less than 2 s were removed from analysis. Time spent exploring the objects was recorded during the choice phase. The preference index was quantified as follows: preference index = (novel object exploration time − familiar object exploration time)/(novel object exploration time + familiar object exploration time).

### sn-RNAseq

sn-RNAseq was performed using WT (7-mo-old; three mice), GKO (7-mo-old; three mice), J20;WT (6-mo-old; one mouse), and J20;GKO (6-mo-old; one mouse) mice. Single nuclei were isolated based on a previously published protocol (Krishnaswami et al., 2016), with modifications. After dissection, hippocampi were homogenized in a cold Dounce homogenizer. The homogenate was centrifuged at 1,000 *g* for 8 min at 4°C to pellet nuclei. Nuclei were resuspended in 1 ml 29% iodixanol buffer and centrifuged at 13,500 *g* for 20 min at 4°C. Supernatant and floating myelin were removed after centrifugation. Nuclei were resuspended in 100 µl nuclei storage buffer and filtered using a 40-µm cell strainer. Nuclei were stained with trypan blue and counted using a hemocytometer. Nuclei (5,000 from each sample) were used for single-nuclei library preparation using the 10X Genomics platform according to the manufacturer’s protocol. Libraries were sequenced on the NovaSeq platform. Sample demultiplexing, barcode processing, and single-cell counting was performed using the Cell Ranger Single-Cell Software Suite (version 3.0.2; 10X Genomics). Cell Ranger count was used to align samples to the reference genome (mm10). The counting matrix were imported into Seurat package (version 3.0) in R (version 3.6.2) for subsequent analysis. For quality control, nuclei with mitochondrial content >5%, gene number <200, or gene number >7,500 were removed. After filtering, a total of 32,037 individual nuclei across all genotypes were selected for downstream analysis. Data were normalized using a scaling factor of 10,000 by default, and then unique molecular identifier counts were normalized using regularized negative binomial regression. Before integration of the eight samples, 3,000 genes were selected by using SelectIntegrationFeatures as the anchor features. The principal component analysis for the integrated dataset were performed using the first 30 principal components and t-distributed stochastic neighbor embedding analysis was performed with the top 30 PCAs. Clustering was performed using a resolution of 0.8. The raw counting matrix from the Cell Ranger count were subjected to dimensionality reduction using a zero-inflated negative binomial regression model with gene and cell-level covariates. Differential expression of genes between conditions was assessed using DESeq2. The excitatory neurons were selected from the whole single-cell dataset according to the cell annotation. The same process was performed on the Excitatory Neuron dataset but with the cluster resolution parameter set as 0.03 in the Seurat package. WGCNA analysis was performed with default parameters on the matrix of raw counts of the excitatory neurons and the trait information (genotype). Significant DEGs were used for GO biological pathway analysis using EnrichR (Chen et al., 2013). Meta-enrichment analyses were performed using Metascape. Raw and processed sequencing data reported in this paper are available under GEO accession no. GSE157985.

### Cell culture and transfection

Mouse N2a neuroblastoma and N2a695 (overexpressing APP695) cells were grown in medium containing 50% DMEM and 50% Opti-MEM, supplemented with 5% FBS and 200 µg/ml G418 (for N2a695; Life Technologies). HEK293T cells (ATCC; CRL-11268) and HEK293-APP WT and GKO cells were grown in DMEM containing 10% FBS (Wong et al., 2019). SH-SY5Y WT and GSAP KO cells were grown in DMEM-F12 medium containing 10% FBS (Wong et al., 2019). CAD (mouse catecholaminergic neuronal) cells were grown in DMEM-F12 medium containing 8% FBS (Qi et al., 1997). Lipofectamine 2000 and 3000 (Life Technologies) were used for all transient transfections following the manufacturer’s instructions. Myc-Flag–tagged Arcn1 (RC210778), PHB (RC201229), and Erlin2 (RC221700) were obtained from OriGene. Mouse full-length GSAP with HA tag (EX-Mm30424-M07), human GSAP plasmids (EX-Z2830-M07), human GSAP-16k with HA tag (aa 733–854 subcloned from full-length HA-GSAP), Fe65 with Flag tag (EX-M0439-M12), and Fe65 with mCherry tag (EX-Mm20316-M56) were obtained from Genecopoeia. GFP-PP1γ (gift from Angus Lamond and Laura Trinkle-Mulcahy, University of Dundee, Dundee, Scotland; Addgene #44225) and pSpCas9(BB)-2A-Puro (gift from Feng Zhang, Broad Institute, Cambridge, MA; Addgene #48139) were obtained from Addgene. APP-GFP plasmid (full-length human APP695 tagged with GFP at its C terminus) was generated in our laboratory previously (Bettayeb et al., 2016a). Flag-tagged APP-C99 plasmid (Flag appended to the C99 C terminus) was a gift from Wenjie Luo, Weill Cornell Medical College, New York, NY. Mouse GSAP siRNA, human GSAP siRNA, and negative control siRNA were obtained from Dharmacon (On-TARGET plus J-056450-11, LQ-025410-02-0005, and D-001830-02-05).

### SDS-PAGE immunoblotting and IP

Cells were collected and washed with PBS and then lysed with either 3% SDS or radioimmunoprecipitation assay lysis buffer supplemented with protease inhibitor cocktail. Bicinchoninic acid assay was used to determine protein concentration. Equal amounts of protein were subjected to SDS-PAGE using either 10–20% Tris-HCl or 4–12% Bis-Tris precast gels. Proteins were transferred onto a polyvinylidene difluoride (PVDF) membrane, blocked in 5% nonfat milk for 1 h at room temperature and incubated with primary antibodies at 4°C overnight. The following primary antibodies were used: Psen1-CTF antibody (1:1,000, MAB5232; EMD Millipore), APP C-terminal antibody (1:4,000, RU369, in house), APP N-terminal antibody (1:1,000, 14–9749-82; Thermo Fisher Scientific), β-amyloid antibody 6E10 (1:500, 803001; BioLegend), phospho-APP (Thr668) antibody (1:1,000, 3823S; Cell Signaling Technology), GSAP antibody (1:1,000, AF8037; R&D Systems), GSAP antibody (1:1,000, PA5-21092, Thermo Fisher Scientific), PP1β antibody (1:1,000, 07–1217; EMD Millipore), Fe65 antibody (1:1,000, ab91650; Abcam), Fe65 antibody (1:1,000, sc-19751; Santa Cruz Biotechnology), Psen1-NTF antibody (1:1,000, ab71181; Abcam), Erlin2 antibody (1:500, 2959S; Cell Signaling Technology), Vadc1 antibody (1:1,000, ab14734; Abcam), Flag M2 antibody (1:1,000, F3165; Sigma), HA antibody (1:1,000, A190-108A; Bethyl), GAPDH antibody (1:500, sc-365062; Santa Cruz Biotechnology), β-tubulin antibody (1:2,000, ab6046; Abcam), and GFP antibody (1:1,000, ab183734; Abcam). Primary antibodies were detected using HRP-linked secondary antibodies together with Western Lightning Plus-ECL (Perkin Elmer). Fiji (ImageJ) was used to quantify band intensity.

For IP experiments, cell pellets were washed with PBS before lysing in IP lysis buffer (50 mM Tris-HCl, 150 mM NaCl, 1% CHAPSO, pH 7.4, supplemented with protease inhibitor cocktail, and PhosStop), for 10 min on ice. Lysates were then centrifuged at 13,000 *g* for 10 min at 4°C. Prior to IP, supernatants were collected and diluted in IP lysis buffer to reach a CHAPSO final concentration of 0.25%. Primary antibody or IgG control was incubated with lysates overnight at 4°C with tumbling. The next day, 30 µl protein G magnetic beads (Thermo Fisher Scientific) was added into samples for 2-h incubation at 4°C. Protein G magnetic beads were collected and washed four times with lysis buffer containing 0.25% CHAPSO. Immunoprecipitated proteins were eluted with SDS sample buffer supplemented with reducing reagent. Samples were heated at 70°C for 10 min before subjecting to immunoblot analysis.

### MS for binding protein identification

HA or GSAP antibody was covalently conjugated to Dynabeads M-270 Epoxy beads (#14301; Thermo Fisher Scientific) using the antibody coupling kit (#14311D; Thermo Fisher Scientific). Cells cultured in triplicate were lysed in 1% CHAPSO IP lysis buffer (50 mM Tris-HCl, 150 mM NaCl, pH 7.4, supplemented with protease inhibitor cocktail, and PhosStop) and diluted in IP lysis buffer to reach a CHAPSO final concentration of 0.25%. HA or GSAP antibody-conjugated beads were added into the lysate to tumble for 2 h at 4°C. Magnetic beads were then collected and washed three times with 0.25% CHAPSO IP lysis buffer and three times with PBS. The immunoprecipitates were eluted with 8 M urea. Proteins were digested overnight with Endopeptidase Lys-C and trypsin. Peptides were analyzed by nano–liquid chromatography (nano-LC) MS/MS. Data were processed using MaxQuant. Comparing bait versus control samples, a differentially enriched protein was labeled as a GSAP-binding protein candidate when it had either an average difference >1.5 or P value < 0.05. Candidates were subjected to GO biological pathway analysis using DAVID 6.8 (https://david.ncifcrf.gov/). Meta-enrichment analyses were performed using Metascape (Zhou et al., 2019).

### Y2H

Y2H screening was performed using the mating strategy with two *Saccharomyces cerevisiae* strains of opposite mating types (strains CG1945 and Y187) as explained elsewhere (Flajolet et al., 2008). The C terminus of human GSAP cDNA fragment (amino acids from position 497–854) was subcloned in frame with the GAL4-DNA-BD moiety into a pAS2 vector as the bait following standard procedures. The bait construct expression was evaluated prior screening by Western blotting analysis (anti-GAL4 domain antibody) after transfection of the bait plasmids in yeast. Toxicity and autoactivity levels of the bait were also evaluated. A commercial cDNA human brain library (subcloned into pACTII) was used and served as the prey. Plasmids of positive clones growing on selective medium were rescued and submitted to DNA sequencing for clone identification using NCBI-BLAST.

### ELISA for Aβ

Aβ quantification was performed as described in our previous publication (Bettayeb et al., 2016a). Briefly, WT and Fe65KO CAD cells were transiently transfected with APP constructs. Media were replaced 6 h before collecting supernatants. Conditioned media from CAD cells were then diluted in buffer for Aβ measurement following the manufacturer’s instructions (Thermo Fisher Scientific). Aβ levels were normalized to total APP protein levels. For the in vivo experiments, soluble Aβ was extracted from the hippocampi of 19-mo-old mice following an established protocol (Casali and Landreth, 2016). Aβ levels were measured and normalized to total protein levels.

### MAM subcellular fractionation

MAM subcellular fractionation was performed as previously described (Lewis et al., 2016). Briefly, cells were homogenized in a sucrose buffer (0.25 M sucrose) using a Teflon glass homogenizer. Homogenates were centrifuged at 600 *g* for 5 min. Pellets were resuspended in isolation medium (5 mM Hepes, pH 7.4, 250 mM mannitol, and 0.5 mM EGTA) and centrifuged at 10,300 *g* for 20 min. Supernatants were centrifuged at 100,000 *g* for 1 h to separate the microsome and cytosol fractions. Pellets were resuspended in isolation medium, layered on top of a Percoll medium, and centrifuged at 95,000 *g* for 30 min. MAM and mitochondria fractions were collected from different layers after centrifugation. The MAM fraction was centrifuged at 100,000 *g* for 1 h to obtain MAM.

### Immunofluorescence microscopy

For immunofluorescence microscopy, cells were fixed in 4% paraformaldehyde for 10 min. For GSAP and Facl4 staining, fixed CAD cells were permeabilized with 0.5% Triton X-100 and exposed to PBS containing 4% BSA for 1 h. Primary antibodies against GSAP (1:100, AF8037; R&D Systems) and Facl4 (1:100, PA5-27137; Thermo Fisher Scientific) were diluted in PBS containing 1% BSA and 0.1% Triton X-100. Isotype-specific secondary antibodies (1∶2,000) conjugated to Alexa Fluor 488 and Alexa Fluor 594 were used (Xu et al., 2013; Xu et al., 2014). Cells were mounted using Vectashield mounting medium with DAPI (Vector Laboratories) and analyzed using a Zeiss LSM710 Fluorescence Microscope. For live-cell imaging, super-resolution images were acquired using a Zeiss LSM 800 confocal microscope equipped with Airyscan module (Zeiss). Fluorescence was collected with ×40 objective lens. Vesicle trafficking velocity and diffusion coefficient were calculated by MATLAB.

### EM

Cells grown on ACLAR film were fixed with 4% formaldehyde and 2% glutaraldehyde in 0.1 M sodium cacodylate buffer (pH 7.4). Subsequently, cells were washed in buffer, post-fixed with 1% osmium tetra-oxide for 1 h, stained en bloc with 1% uranyl acetate for 30 min, dehydrated by a graded series of ethanol, infiltrated with a resin (Eponate12; Electron Microscopy Sciences), and embedded with the resin. After polymerization at 60°C for 48 h, ultra-thin sections were cut, underwent post-staining with 2% uranyl acetate and 1% lead citrate, and were examined under a JEOL 1400Plus transmission electron microscope.

### Generation of Fe65KO CAD line

A guide RNA sequence (5′-ACG​GAT​TCC​GAT​CTA​CCG​GC-3′) targeting the mouse Fe65 gene was cloned into the pSpCas9(BB)-2A-Puro vector, a gift from Feng Zhang (plasmid #48139; Addgene). The plasmid was transfected into CAD cells, which underwent 1 µg/ml puromycin selection 48 h after transfection. Cells were seeded in clonal limiting dilution in 96-well plates. Fe65KO cells were screened and validated by immunoblot analysis and Sanger sequencing.

### Lipidomics analysis

Lipid extracts were prepared using a modified Bligh and Dyer method (Bligh and Dyer, 1959). Extracts were spiked with appropriate internal standards and analyzed by LC/MS as described (Chan et al., 2012). Briefly, glycerophospholipids and sphingolipids were separated with normal-phase HPLC, while sterols and glycerolipids were separated with reverse-phase HPLC using an isocratic mobile phase. Individual lipid species were quantified by referencing to spiked internal standards. The nomenclature abbreviations are listed in Fig. S5 B.

### Lipid overlay assay

A nitrocellulose membrane spotted with the indicated lipids (Echelon Biosciences) was blocked in 3% BSA in PBST (0.1% Tween 20) at 4°C overnight. HEK293T cells transiently expressing HA-GSAP were lysed in 0.5% Triton lysis buffer (50 mM Tris-HCl and 150 mM NaCl, pH 7.4). Cell lysate (200 µg) was diluted in 3% BSA in PBST and then incubated for 1 h with the membrane at room temperature. After washing, GSAP association with lipids was detected using HA antibody and HRP-conjugated secondary antibody.

### Cellular respiration analysis

Oxygen consumption reflecting mitochondrial activity was measured by XF mito stress kit according to the manufacturer’s protocol. All the measurements were performed using an Agilent Seahorse XFe96 analyzer from the High-Throughput and Spectroscopy Resource Center. WT and GKO SHSY-5Y cells were seeded at 20,000 per well in a 96-well plate 1 d before the measurement. OCR was measured after sequential addition of 1 µM oligomycin, 1 µM FCCP and 0.5 µM rotenone/antimycin. The results were analyzed using the Wave software (Agilent) and normalized by the cell number, which was measured by the ImageXpress-micro system.

### Identification and annotation of AD-associated genetic variants in GSAP locus

AD association of GSAP variants (GRCh37, chromosome 9: 76,890,110–77,095,630) was obtained from recently published AD genome-wide association studies summary statistics (Jansen et al., 2019). GSAP variants with P < 0.05 were retained as variants exerting AD association. The obtained AD-associated GSAP variants were subjected to the SCREEN hg19 database (https://screen-v10.wenglab.org/gwasApp/?assembly=hg19; Moore et al., 2020) for annotating variants that may reside in the candidate cis-regulatory elements. IGV (Integrative Genomics Viewer; version 2.8.7) was used to visualize the epigenic events in rs74338692-assocated genomic regions. Specifically, the following datasets were analyzed in the study: cell type–agnostic ccRE (ENCODE ID: ENCFF788SJC); monocytes: DNase-seq (ENCODE ID: ENCFF398USK), H3K27ac (ENCODE ID: ENCFF931PZJ), and H3K4me1 (ENCODE ID: ENCFF731YSQ); bipolar neurons: DNase-seq (ENCODE ID: ENCFF106BSM) and H3K27ac (ENCODE ID: ENCFF967OEW); transcription factor–binding events: FOSL2 (ENCODE ID: ENCFF321KVH).

### Statistical analysis and data visualization for GSAP variants

Linkage disequilibrium and haplotype analysis for six GSAP variants residing in the candidate cis-regulatory elements were conducted using 1000 Genomes Project phase 3 whole-genome sequencing data of the European Super Population (*n* = 503). In brief, genotypes for those six SNPs stored in VCF files were extracted and subjected to PLINK (version v1.90b6.12; Purcell et al., 2007) for analysis. The ped file obtained from PLINK analysis was subsequently subjected to Haploview (version 4.2; Barrett et al., 2005) for linkage disequilibrium and haplotype analysis and visualization. For genotype-expression association analysis, the whole-genome sequencing genotype information obtained from the GTEx (phs000424.v8.p2) was further subjected to BEAGLE (version r1399) for haplotype phasing (nthreads = 24, phase-its = 50, impute-its = 30). The haplotypes constructed by six variants were obtained by R programming analysis of phased genotypes. Association analysis was conducted between GSAP variant or haplotype dosage and GSAP transcript levels in 13 brain regions recorded in GTEx database by robust regression analysis (R robustbase packages). Meta-analysis was further performed by summarizing results at the tissue level using METASOFT (version 2.0.1). GraphPad Prism (version 8.0.1) was used to generate bar and dot plots.

### Statistical analysis

Statistical analysis was performed using GraphPad Prism. Results are presented as mean ± SEM or mean ± SE as indicated. MATLAB was used for live-cell imaging data analysis. Two-tailed unpaired Student’s *t* test was used, except for sn-RNAseq analysis. P < 0.05 was considered significant (*, P < 0.05; **, P < 0.01; ***, P < 0.001; and ****, P < 0.0001). For the animal behavior study, mice from the same litter were randomized into groups, and the experiment was performed blinded to genotype.

### Online supplemental material

Fig. S1 lists identified GSAP-binding proteins and validation of GSAP antibodies. Fig. S2 shows Aβ level changes after Fe65KO in CAD cells and GKO mouse characterization. Fig. S3 shows sn-RNAseq analysis of DEGs and biological pathway changes in additional cell types. Fig. S4 shows abbreviations of lipid species and lipid–GSAP interaction. Fig. S5 shows GSAP transcript level increases during aging (PsychENCODE) and lists of candidate SNPs affecting GSAP expression in human brain tissues.
